# Trap diversity and character evolution in carnivorous bladderworts (*Utricularia*, Lentibulariaceae)

**DOI:** 10.1038/s41598-017-12324-4

**Published:** 2017-09-21

**Authors:** Anna Sofia Westermeier, Andreas Fleischmann, Kai Müller, Bastian Schäferhoff, Carmen Rubach, Thomas Speck, Simon Poppinga

**Affiliations:** 1 Plant Biomechanics Group, Botanic Garden, University of Freiburg, Schänzlestraße 1, D-79104 Freiburg im Breisgau, Germany; 2 Freiburg Center for Interactive Materials and Bioinspired Technologies (FIT), University of Freiburg, Georges-Köhler-Allee 105, D-79110 Freiburg im Breisgau, Germany; 3 Botanische Staatssammlung München, Menzinger Straße 67, D-80638 München, Germany; 4 GeoBio-Center LMU, Center of Geobiology and Biodiversity Research, Ludwig-Maximilians-University, München, Germany; 5 Westfälische Wilhelms-Universität Münster, Institut für Evolution und Biodiversität, AG Evolution und Biodiversität der Pflanzen, Hüfferstraße 1, D-48149 Münster, Germany; 6 PAN Institut für Endokrinologie und Reproduktionsmedizin, Zeppelinstraße 1, D-50667 Köln, Germany

## Abstract

Bladderworts (*Utricularia*, Lentibulariaceae, Lamiales) constitute the largest genus of carnivorous plants but only aquatic species (about one fifth of the genus) have so far been thoroughly studied as to their suction trap functioning. In this study, we comparatively investigated trap biomechanics in 19 *Utricularia* species to examine correlations between life-forms, trapping mechanisms, and functional-morphological traits. Our investigations show the existence of two functional trap principles (passive trap in *U*. *multifida* vs. active suction traps), and – in active suction traps – three main trapdoor movement types (with several subtypes). The trapdoor movement types and their corresponding functional-morphological features most presumably represent adaptations to the respective habitat. We furthermore give insights into fluid dynamics during suction in three representatives of the main types of trapdoor movement. The results on functional morphology and trapdoor movement were mapped onto a new phylogenetic reconstruction of the genus, derived from the rapidly evolving chloroplast regions *trnK*, *rps16* and *trnQ-rps16* and a sampling of 105 *Utricularia* species in total. We discuss potential scenarios of trap character evolution and species radiation, highlighting possible key innovations that enable such a unique carni﻿vorous lifestyle in different habitats.

## Introduction

Bladderworts (*Utricularia*) belong to the most recently evolved group of carnivorous plants, the Lentibulariaceae within the order Lamiales^1^. The genus draws attention not only because some of its species have one of the smallest genomes known from angiosperms with around 80 Mbp^2–4^ but also by developing ultra-fast suction traps (‘bladders’). As established by morphological and molecular systematic studies, it comprises 32 generic sections in three subgenera^5–9^. High DNA substitutional rates in various genomic regions correlate with an astounding morphological and taxonomic diversity (ca. 240 species, different lifeforms)^5,7,10–12^. The largest part of the genus (ca. 84%) comprises terrestrial species in the widest sense (~being affixed to the soil, including epiphytic and lithophytic species), and ca. 16% are free-floating aquatics. Although we already have a good understanding on how traps work physiologically and mechanically in aquatic species (as concisely described in the following, and reviewed by ref.^13^), almost nothing is known about traps from species with other life-forms.

Generally, suction is based on energy-demanding water pumping processes from the trap lumen to the outside, thereby generating a lower hydrostatic pressure inside the trap^14^ and leading to deformation of the lateral, flexible trap walls that store elastic energy. The motile trapdoor seals the trap watertight with the aid of mucilage and a fringe-like epithelial structure termed velum^13,15,16^. Its free lower edge fits into a cavity situated along the threshold. In the ready-to-catch state, the door possesses an outward, convex curvature^17^. When prey touches the trigger hairs, the trapdoor abruptly inverts its curvature to concave (when seen from the outside), which is hypothesized to be facilitated by concentric cellular constrictions on its inner surface^17^. Then, it swings open, the trap walls relax, and water and prey rush into the trap due to the sudden increase of bladder volume, all within only a few milliseconds. Afterwards, the door eventually re-closes by re-inverting its curvature. Fluid sucked into the trap is being accelerated with up to 600 *g*, and a top speed of 1.5 m/s is reached (measured for *U. inflata* ^17^). Spontaneous firings (SFs) (i.e. suction events without stimulation of the trigger hairs) at critical pressure values inside the traps were observed, and three patterns of SFs could be distinguished: random, metronomic, and ‘bursts’ (i.e, many fast and consecutive suction events)^17–20^. These events may lead to an accumulation of pelagic biomass (‘algae’, debris, and detritus) which complements nutrition of *Utricularia* ^21^.

Two *Utricularia* trap types have been distinguished so far^15^. In the ‘*Utricularia vulgaris* trap type’, the free edge of the trapdoor rests on the threshold at an obtuse angle of about 90° (as seen in sagittal section), and the trap entrance is shaped like a short tube. This applies to all species of *Utricularia* sect. *Utricularia* (all of them aquatics). In the second (unnamed) type, to which many non-aquatic species are attributed to, the trap entrance is long and tubular with the door standing obliquely at an acute angle of about 30°.

Because of the fact that water as a constantly surrounding medium enabling for prey-triggered and spontaneous suction may not be permanently present, the question arises whether traps of non-aquatic species function similarly to those of aquatic species, or if functional and/or structural differences exist. Such knowledge, in combination with novel molecular phylogenetic analyses, would allow for drawing conclusions regarding the evolution of functional and structural traits and may help in explaining the evolutionary success of the genus. Therefore, we examined in detail functional principles of traps and trapdoor movements in 18 non-aquatic and one aquatic bladderwort species from 13 of the 32 generic sections. The doors were investigated morphologically with respect to the following functionally important structures: trigger hairs (trap triggering), cavities on the thresholds (door fastening), vela and mucilage (sealing of the trap, positioning of the door), and concentric cellular constrictions on the inner trapdoor surfaces (channelling the curvature inversion). Additionally, comparative biomechanical and morphological analyses were performed, concerning triggered trapping action (suction, trapdoor movement) and SFs without triggering. The resulting data are analysed in the context of a phylogenetic reconstruction of the genus, and possible scenarios of trap character evolution and species radiation are discussed.

## Results

### Trapdoor kinematics and functional trap morphology

**Figure 1:**
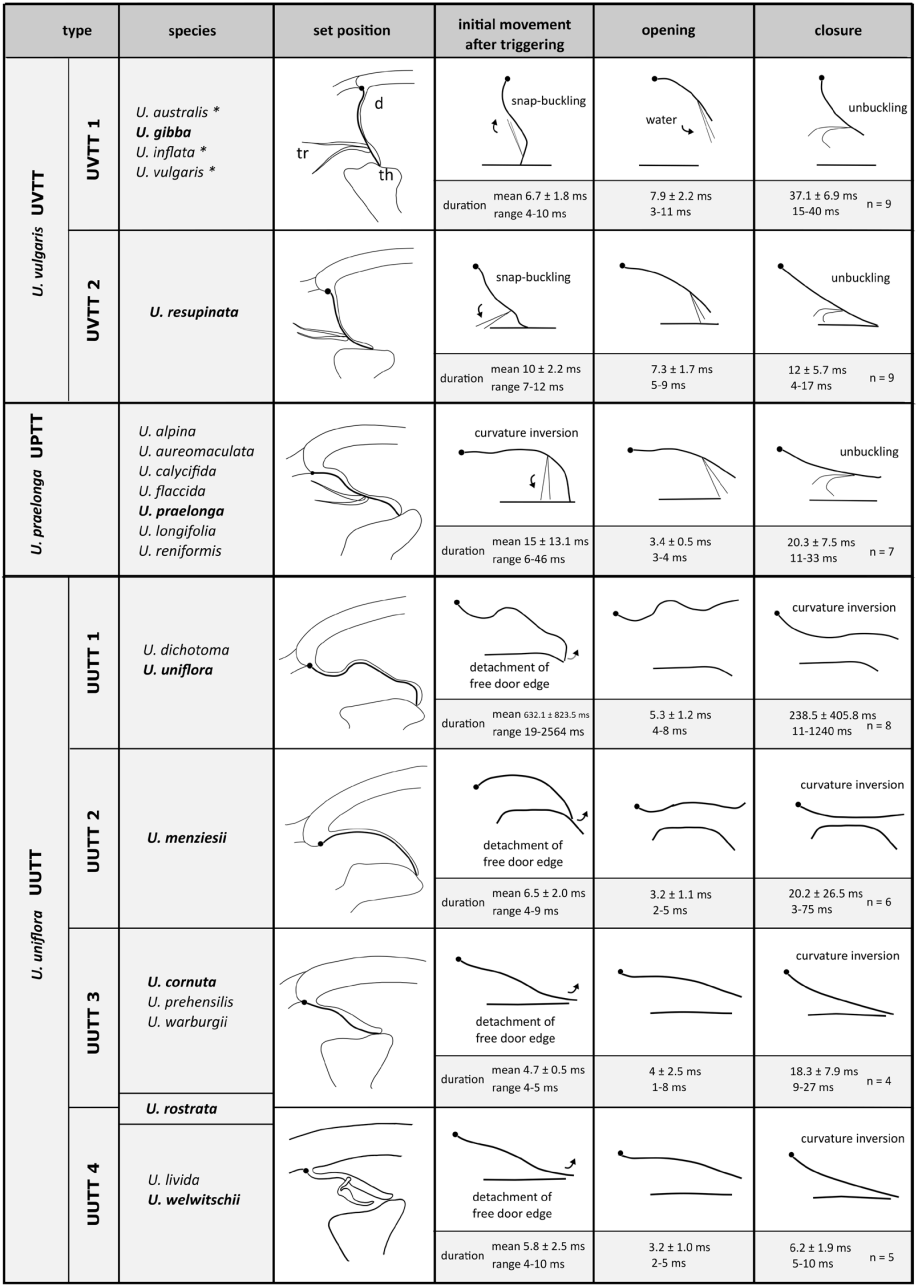
*Utricularia* trapdoor movement types in active traps. Schematic drawings in column ‘set position’ depict sagittal sections of trap entrances, highlighting the initial door postures when the traps are ready to fire. Columns right from ‘set position’ depict different movement steps observed for respective species. Timescales and standard deviations are given for species written in boldface (see Table 2 for detailed results on all species). For species indicated by an asterisk (*) see ref.^17^. The threshold (th) as well as the progression of the median door axis (d) and the movement of trigger hairs (tr) are outlined. In the *U. vulgaris* trapdoor type (UVTT), the trapdoor performs a curvature inversion from convex to concave prior to opening (Movie S1). The UVTT1 is the trapdoor type as present in the ‘*Utricularia vulgaris* trap type’ with the obtuse door-to-threshold angle (90°) (see Introduction). In the UVTT2, the trigger hair movement is different to the UVTT1 (Movie S2). In the *U. praelonga* trapdoor type (UPTT), the door is in acute angle in relation to the threshold and, like in the UVTT, performs curvature inversion prior to opening (Movie S3). Doors of the *U. uniflora* trapdoor type (UUTT) do not perform curvature inversions prior to opening (Movies S4–S7). They slowly detach from the threshold and then swing open. The different subtypes (UUTT1-4) are subdivided according to the divergent door postures in the set positions and to the occurrence of trigger hairs.

High-speed recordings of the manually triggered trapdoors (Movies S1–S7) allowed us to determine trap entrance morphology and trapdoor postures in set positions and to analyse the fast movements during trap suction activity (Fig. 1, Tables 1 and 2). Based on these analyses, we were able to assign the 19 investigated *Utricularia* species to two functional trap principles (active and passive). The 18 species with active traps were further assigned to three main trapdoor types (with several subtypes).

**Table 1:** List of species investigated, generic sections, life-forms, trapdoor types, functional door morphology and observed mode(s) of spontaneous firings (SFs).

Species	Section (sensu ref.^9^)	Subgenus (sensu ref.^5^)	Life-form (sensu ref.^5^)	Trapdoor type	SF	Functional trap morphology					
						Trapdoor angle [deg°]	Trigger hairs or similar structures	Concentric constrictions	Cavity	Velum	Mucilage
*U. multifida* R.Br.	*Polypompholyx*	*Polypompholyx*	Terrestrial/facultative semiaquatic, annual	no motion	—		Sessile glands	None	None	None	Weak
*U. uniflora* R.Br.	*Pleiochasia*	*Polypompholyx*	Terrestrial, perennial	UUTT1	r, b	24	Sessile glands	None	None	None	Weak
*U. dichotoma* Labill.	*Pleiochasia*	*Polypompholyx*	Terrestrial/facultative semiaquatic, perennial	UUTT1	r	24	Sessile glands	None	None	None	Weak
*U. menziesii* R.Br.	*Pleiochasia*	*Polypompholyx*	Terrestrial, perennial geophyte	UUTT2	observed only once	19	Sessile glands	None	None	None	?
*U. rostrata* A.Fleischm. & Rivadavia	*Aranella*	*Bivalvaria*	Terrestrial, perennial	UUTT3 or 4 (*)	r	?	?	None	?	?	?
*U. warburgii* K.I.Goebel	*Nigrescentes*	*Bivalvaria*	Terrestrial, perennial	UUTT3	r	22	Sessile glands	None		None	
*U. welwitschii* Oliv.	*Calpidisca*	*Bivalvaria*	Terrestrial, perennial	UUTT4	r, b	38	Kriss-trichome		None	None	Distinct
*U. livida* E.Mey.	*Calpidisca*	*Bivalvaria*	Terrestrial, perennial	UUTT4	r	36	Trigger hairs	None	None	None	Distinct
*U. cornuta* Michx.	*Stomoisia*	*Bivalvaria*	Terrestrial/semiaquatic, perennial	UUTT3	r, b	33	None		Weak	None	Distinct
*U. prehensilis* E.Mey.	*Oligocista*	*Bivalvaria*	Facultative semiaquatic/terrestrial, perennial	UUTT3	r, m, b	35	Sessile glands	Weak	Weak	None	Weak
*U. calycifida* Benj.	*Foliosa*	*Utricularia*	Terrestrial, perennial	UPTT	r, b	58	Trigger hairs	Weak	Weak	None	Weak
*U. longifolia* Gardner	*Foliosa*	*Utricularia*	Terrestrial/facultative lithophytic, perennial	UPTT	r, b	62	Trigger hairs	Weak	Weak	None	Weak
*U. praelonga* A.St-Hill. & Girard	*Foliosa*	*Utricularia*	Terrestrial, perennial	UPTT	r	58	Trigger hairs	Weak	Weak	None	Distinct
*U. alpina* Jacq.	*Orchidioides*	*Utricularia*	Terrestrial/facultative epiphytic, perennial	UPTT	r	52	Trigger hairs	Weak	Weak	None	Weak
*U. reniformis* A.St-Hill.	*Orchidioides*	*Utricularia*	Terrestrial/facultative epiphytic, perennial	UPTT	r	51	Trigger hairs	Weak	Weak	None	Distinct
*U. resupinata* B.D.Greene ex Bigelow	*Leticula*	*Utricularia*	Affixed subaquatic, perennial	UVTT2	r	92	Trigger hairs	Weak	Weak	Distinct	?
*U. flaccida* A.DC.	*Setiscapella*	*Utricularia*	Terrestrial, perennial	UPTT	r	37	Trigger hairs	Weak	Weak	None	Distinct
*U. gibba* L.	*Utricularia*	*Utricularia*	Aquatic, perennial	UVTT1	r, b	91	Trigger hairs	Distinct	Distinct	Distinct	?
*U. aureomaculata* Steyerm.	*Steyermarkia*	*Utricularia*	Terrestrial, facultative lithophytic, perennial	UPTT	r	46	Trigger hairs	Weak	Weak	None	Distinct

Abbreviations: b: burst; m: metronomic; r: random. UPTT = *U. praelonga* trapdoor type; UUTT = *U. uniflora* trapdoor type; UVTT = *U. vulgaris* trapdoor type. In fields marked with a “?”, the respective character could not be assessed. (*) Due to lack of data on trigger hairs, the exact subtype could not be evaluated in *U. rostrata*.

**Table 2:** Durations of different movement steps of the respective trapdoor movement types.

Species & number of traps investigated (n)	Trapdoor type	Duration of initial trapdoor movement after triggering [ms]			Trapdoor opening duration [ms]			Trapdoor closure duration [ms]		
		min	max	mean and standard deviation	min	max	mean and standard deviation	min	max	mean and standard deviation
*U. uniflora*, n = 8	UUTT1	19	2564	632.1 ± 823.5	4	8	5.3 ± 1.2	11	1240	238.5 ± 405.8
*U. dichotoma*, n = 3	UUTT1	5	19	11 ± 5.9	2	5	4 ± 1.4	12	75	42.3 ± 25.8
*U. menziesii*, n = 6	UUTT2	4	9	6.5 ± 2.0	2	5	3.2 ± 1.1	3	75	20.2 ± 26.5
*U. rostrata*, n = 1	UUTT3 or 4	Included in trapdoor opening duration			6			10		
*U. warburgii*, n = 6	UUTT3	5	74	24.2 ± 26.9	3	5	3 ± 1.5	2	36	12 ± 11.4
*U. welwitschii*, n = 5	UUTT4	4	10	5.75 ± 2.5	2	5	3.2 ± 1.0	5	10	6.2 ± 1.9
*U. livida*, n = 3	UUTT4	2	3	2.7 ± 20.5	2	2	2 ± 0.0	7	12	10.3 ± 2.4
*U. cornuta*, n = 4	UUTT3	4	5	4.7 ± 0.5	1	8	4 ± 2.5	9	27	18.3 ± 7.9
*U. prehensilis*, n = 10	UUTT3	1	29	11.4 ± 7.2	3	6	4.1 ± 1.0	8	21	13.4 ± 4.0
*U. calycifida*, n = 7	UPTT	5	7	6.4 ± 0.7	2	3	2.7 ± 0.5	10	26	16.7 ± 4.9
*U. longifolia*, n = 7	UPTT	6	7	6.5 ± 0.5	2	3	2.1 ± 0.3	12	20	15.3 ± 3.0
*U. praelonga*, n = 7	UPTT	6	46	15 ± 13.1	3	4	3.4 ± 0.5	11	33	20.3 ± 7.5
*U. alpina*, n = 1	UPTT	12	12	12	3	3	3	6	6	6
*U. reniformis*, n = 3	UPTT	4	18	9 ± 6.4	3	4	3.7 ± 0.5	20	52	38.3 ± 13.5
*U. resupinata*, n = 3	UVTT2	7	12	10 ± 2.2	5	9	7.3 ± 1.7	4	17	12 ± 5.7
*U. flaccida*, n = 2	UPTT	7	11	9 ± 2.0	2	3	2.5 ± 0.5	6	21	13.5 ± 7.5
*U. gibba*, n = 9	UVTT1	4	10	6.7 ± 1.8	3	11	7.9 ± 2.2	15	40	27.1 ± 6.9
*U. aureomaculata*, n = 4	UPTT	5	17	8.8 ± 4.8	2	3	2.3 ± 0.4	8	30	22.3 ± 8.6

Abbreviations: UPTT = *U. praelonga* trapdoor type; UUTT = *U. uniflora* trapdoor type; UVTT = *U. vulgaris* trapdoor type. In *U. rostrata*, the duration of initial trapdoor movement after triggering could not be distinguished from the trapdoor opening duration.

### The *U. vulgaris* trapdoor type (UVTT)

**Figure 2:**
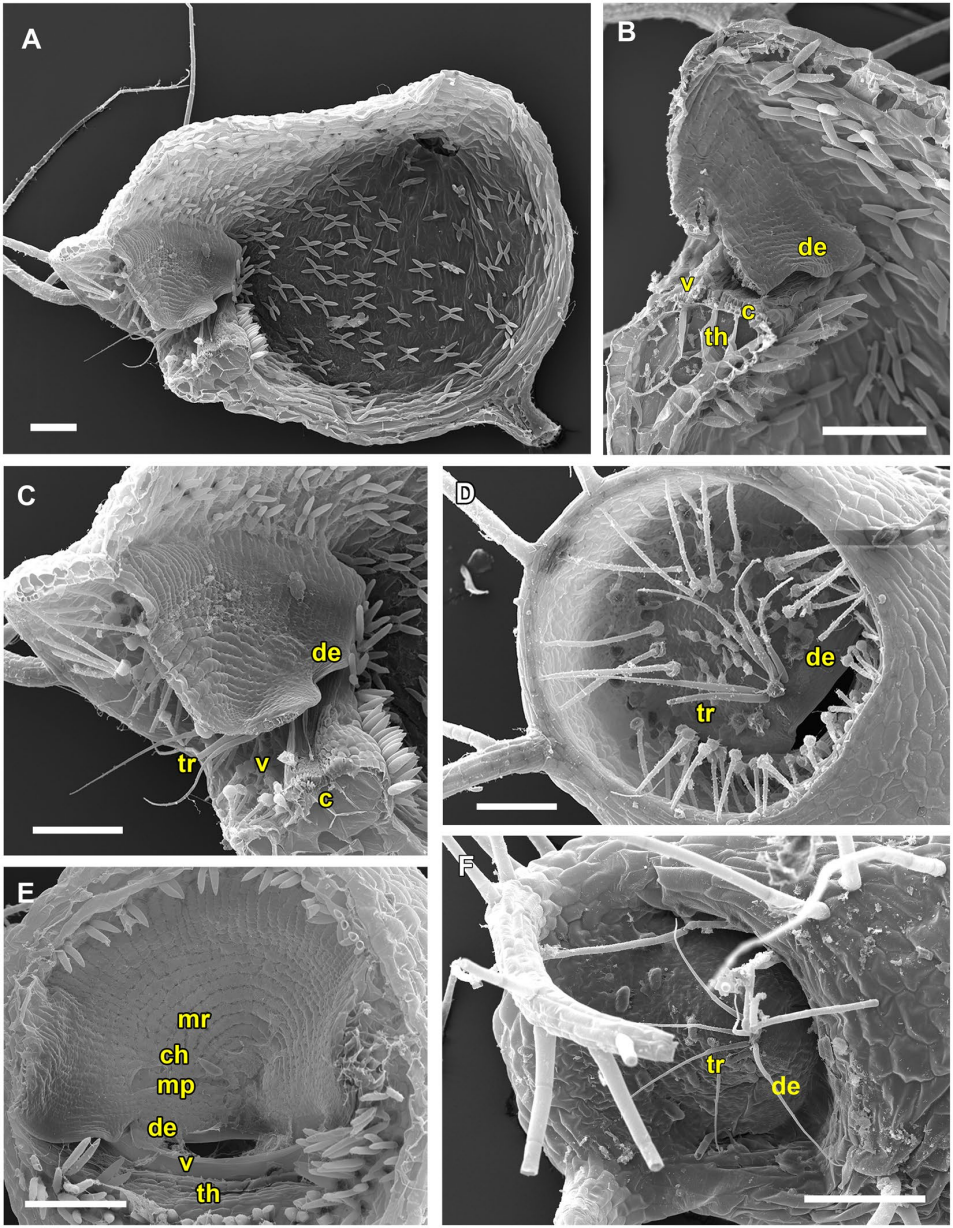
SEM images showing morphological trap characteristics of *U*. *gibba* (representing the UVTT 1) and of *U. resupinata* (representing the UVTT 2). (**A**) Sagittal section of a trap of *U. gibba*. The entrance forms a short tube. The trapdoor, the threshold, and the different glands covering the trap inside are clearly visible. (**B**) Sagittal section of an *U. resupinata* trap entrance. Note the cavity on the threshold, the free door edge, the trigger hairs, the velum and the threshold. (**C**) Exterior view on trap entrance of *U. resupinata*. The trigger hairs, the free door edge and a multitude of glandular structures are visible. (**D**) Sagittal section through the short trap entrance of *U. gibba* with the trigger hairs, the door edge and distinct velum and cavity on the threshold clearly visible. (**E**) Exterior view on trap entrance of *U. gibba*. (**F**) Concentric cellular constrictions on the inner door surface of *U. gibba*, with different door regions sensu [15] indicated. Scale bars 100 µm. Abbreviations: c = cavity, ch = central hinge, de = door edge, mp = middle piece, mr = middle region, th = threshold, tr = trigger hairs, v = velum.

Traps featuring the UVTT are characterized by short entrance regions (Fig. 2A–C). The angles between doors and thresholds are obtuse (~90°) and the trapdoors invert their curvatures from convex to concave prior to opening.

The UVTT can be subdivided into two subtypes. UVTT1 is found in *U. gibba* from *U*. sect. *Utricularia* (Movie S1). The cavity on the threshold and the velum are well visible in the entrance region (Fig. 2B,C). As written above, the free trapdoor edge rests on the threshold at an obtuse angle of approximately 90°. The door bulges outwards (convex curvature) when the trap is ready to fire. Upon stimulation of the trigger hairs, the door inverts its curvature and the hairs flap upwards and against the outer door surface (duration: *U. gibba* mean 6.7 ms, standard deviation ± 1.8 ms, range 4–10 ms, n = 9). Afterwards, the door swings open due to the inflow of water (duration: *U. gibba* mean 7.9 ± 2.2 ms, range 3–11 ms, n = 9). It was observed that the water flow also causes a downwards flapping of the trigger hairs in direction to the trap lumen (streamlining). After suction, the door resets and regains its initial curvature (closure) (duration: *U. gibba* mean 27.1 ± 6.9 ms, range 15–40 ms, n = 9) and the trigger hairs (Fig. 2D) regain their initial orientation. The inner door surface possesses pronounced concentric cellular constrictions (Fig. 2E).

The second subtype, UVTT2, is found in the submerged, affixed aquatic *U. resupinata* of *U*. sect. *Leticula* (Movie S2). Similar to the first subtype, the door-to-threshold angle is obtuse (approximately 90°) and the door possesses a convex curvature which quickly inverts after triggering (duration: mean 10 ± 2.2 ms, range 7–12 ms, n = 3). The only difference to the UVTT1 is that the trigger hairs (Fig. 2F) do not flap upwards onto the outer door surface after triggering, but downwards. The door opens with the trigger hairs still pointing downwards (i.e. already streamlined) (duration: mean 7.3 ± 1.78 ms, range 5–9 ms, n = 3) and resets via reverse curvature inversion (duration: mean 12 ± 5.7 ms, range 4–17 ms, n = 3). Hereby, the trigger hairs regain their initial orientation.

### The *U. praelonga* trapdoor type (UPTT)

**Figure 3:**
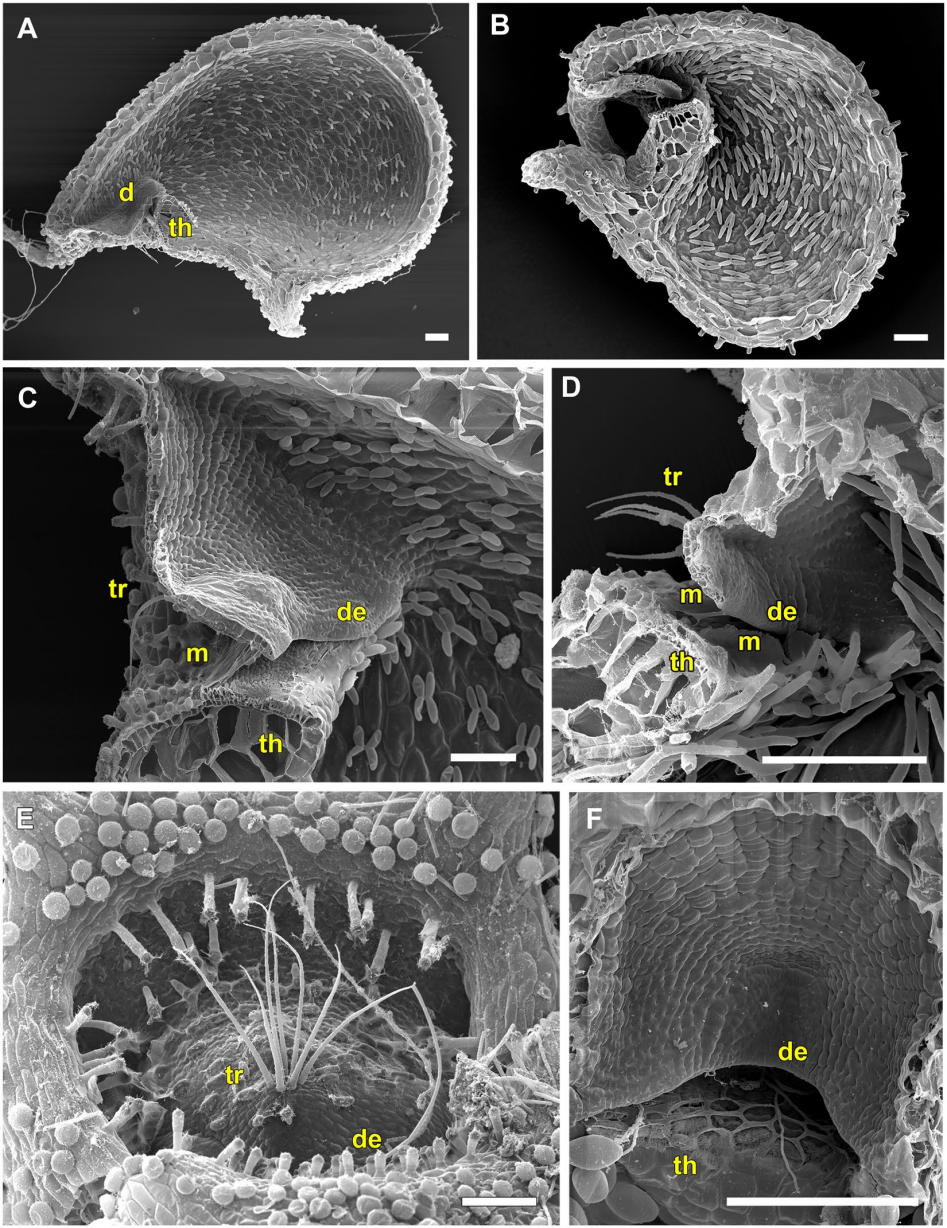
SEM images showing morphological trap characteristics of *U. reniformis*, *U. alpina* and *U. flaccida*, all representing the UPTT. (**A**) Sagittal section through an *U. reniformis* trap with the tubular trap entrance visible. (**B**) Sagittal section of an *U. alpina* trap. The narrow trap entrance is tube-shaped with the appendages covering the entrance. (**C**) Sagittal section through the *U. reniformis* trap entrance. Clearly visible are the trigger hairs protruding from the trapdoor, the free door edge, the threshold and the mucilage on the threshold. (**D**) Sagittal section through an *U. flaccida* trap entrance. (**E**) View on the outer surface of an *U. reniformis* trapdoor with trigger hairs. (**F**) View on the inner surface of the trapdoor of *U. reniformis* with a weak concentric cellular constriction pattern. Scale bars = 100 µm. Abbreviations: de = door edge, m = mucilage, th = threshold, tr = trigger hairs.

**Figure 4:**
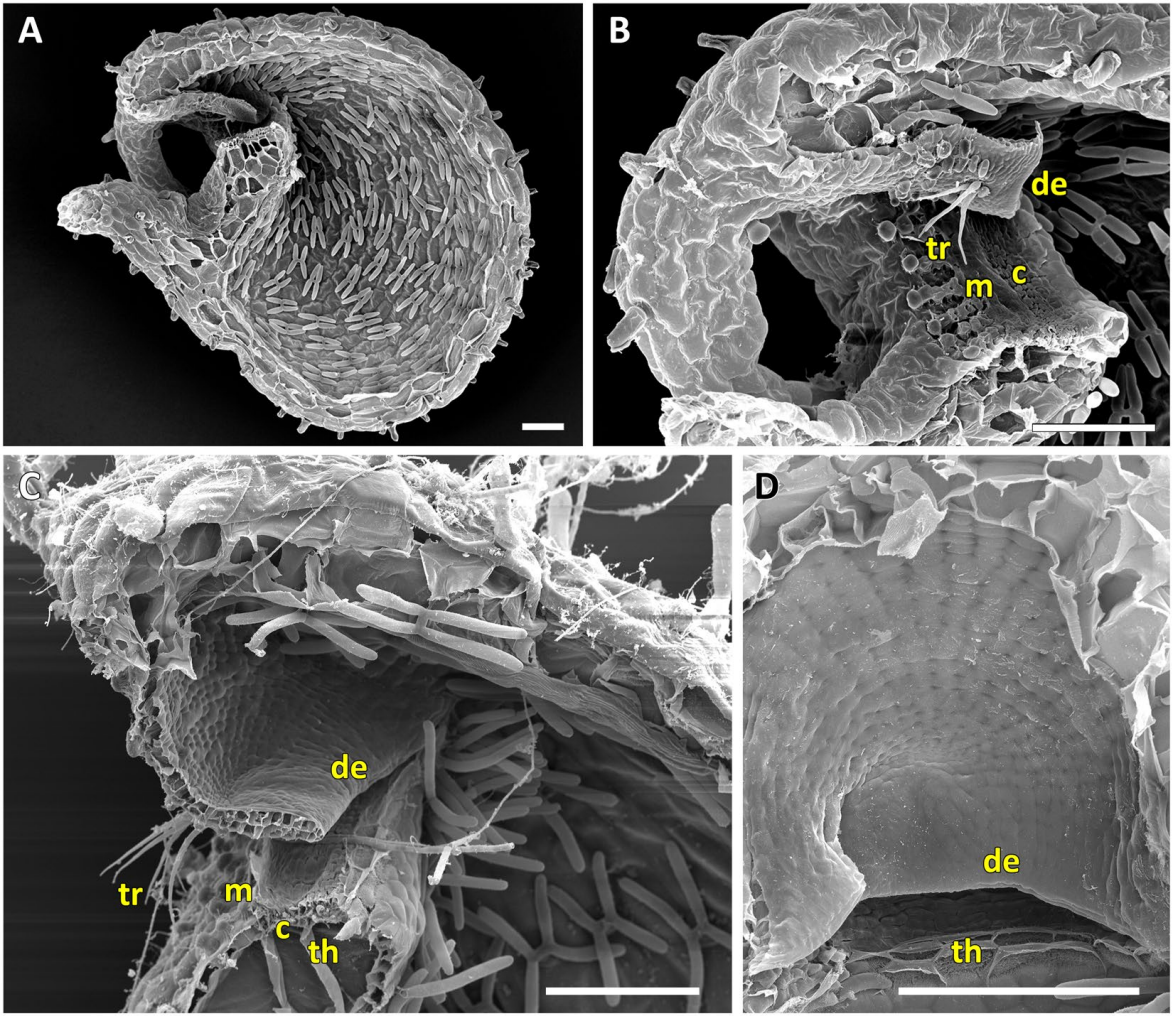
SEM images of *U. alpina* and *U. aureomaculata*, representing the UPTT. (**A**) Sagittal section of a trap of *U. alpina*. The narrow trap entrance is shaped like a tube, the appendages covering the entrance are clearly visible. (**B**) Sagittal section through trap entrance of *U. alpina*. Clearly visible are the trigger hairs, the free door edge, the cavity, and mucilage sticking to the threshold. (**C**) Sagittal section through a trap entrance with a rather tubular shape of *U. aureomaculata*. Clearly visible are the trigger hairs, the free door edge, the threshold and the mucilage on the threshold. A shallow cavity on the threshold can be observed. (**D**) View on the inner surface of trap door of *U. aureomaculata*. A distinct cellular constriction pattern cannot be identified. Scale bars = 100 µm. Abbreviations: c = cavity, de = door edge, m = mucilage, th = threshold, tr = trigger hairs.

**Figure 5:**
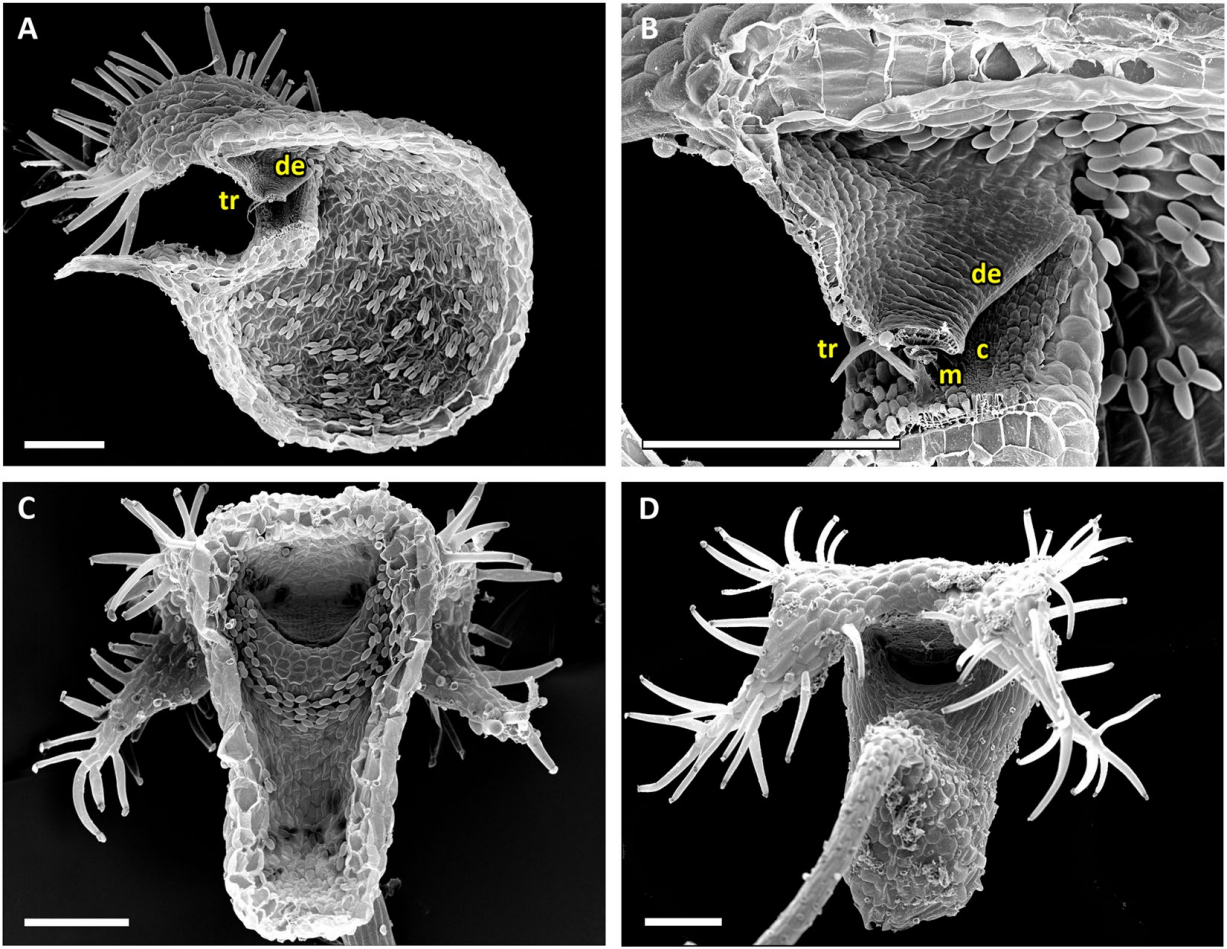
SEM images of a trap, the trap entrance and trapdoor of *U. calycifida*, representing the UPTT. (**A**) Sagittal section of a trap. The tubular shape of the trap entrance, the free door edge and the protruding trigger hairs are visible. (**B**) Sagittal section through the trap entrance. Visible are the free door edge, the trigger hairs, a weak cavity on the threshold and mucilage between the door and the threshold. (**C**) Interior view on the front part of a bladder. (**D**) Frontal, exterior view on an *U. calycifida* trap. Note the filamentous appendages covering the obscured, narrow trap entrance. Scale bars = 200 µm. Abbreviations: c = cavity, de = door edge, m = mucilage, th = threshold, tr = trigger hairs.

**Figure 6:**
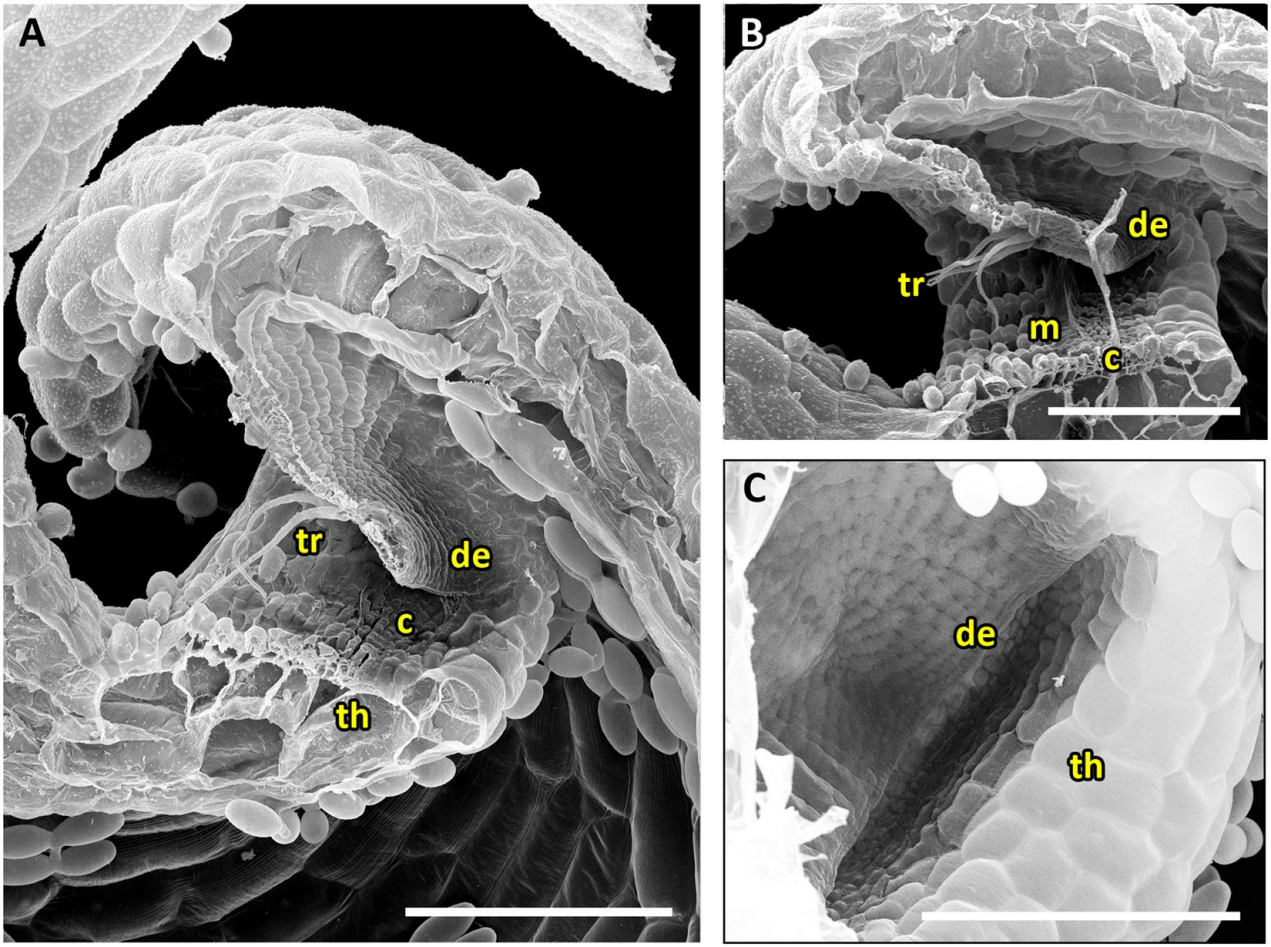
SEM images of trap entrance and trapdoor of *U. longifolia* representing the UPTT. (**A** and **B**) Sagittal sections through the tubular trap entrance. Clearly visible are the trigger hairs, the free door edge and a weak cavity on the threshold. In **B**) also mucilage sticking to the threshold can be seen. (**C**) View on the inner door surface. A pattern of concentric constrictions is faintly visible. Scale bars = 100 µm. Abbreviations: c = cavity, de = door edge, m = mucilage, th = threshold, tr = trigger hairs.

**Figure 7:**
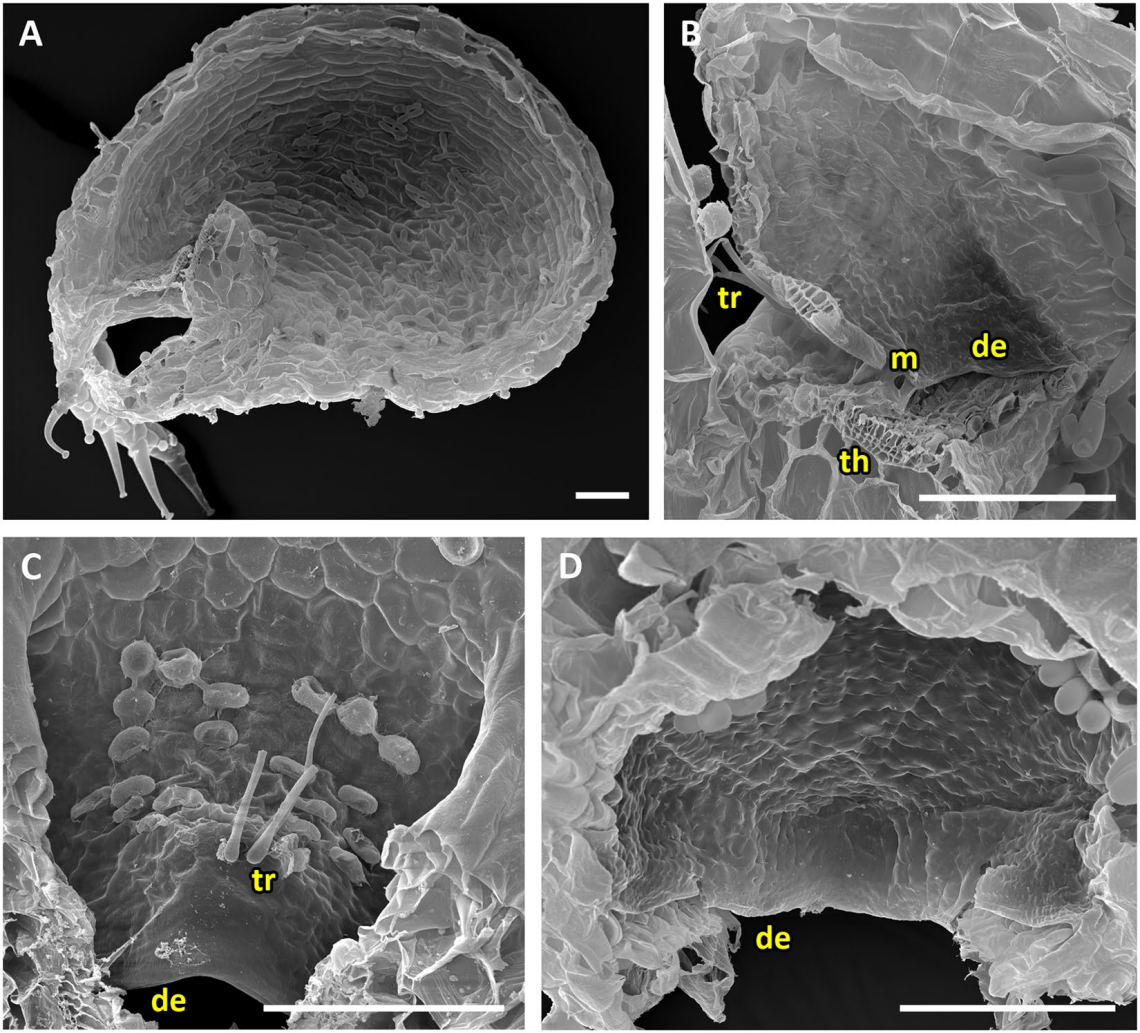
SEM images of a trap, trap entrance and trapdoor of *U. praelonga* representing the UPTT. (**A**) Sagittal section through a trap with the tubular trap entrance visible. (**B**) Sagittal section through the trap entrance. Note the trigger hairs and mucilage sticking to the free door edge and the threshold. (**C**) View on the outer surface of the trapdoor with trigger hairs. (**D**) View on the inner surface of the trapdoor. A distinct concentric cellular constriction pattern is not visible. Scale bars = 100 µm. Abbreviations: de = door edge, m = mucilage, th = threshold, tr = trigger hairs.

The UPTT is found in *U. alpina* and *U. reniformis* (*U*. sect. *Orchidioides*), *U. calycifida*, *U. longifolia* and *U. praelonga* of *U*. sect. *Psyllosperma*, *U. aureomaculata* (*U*. sect. *Steyermarkia*), and *U. flaccida* (*U*. sect. *Setiscapella*) (Movie S3). All traps of the UPTT possess a tubular entrance region (Figs 3A–D, 4A,B, 5A,B, 6A,B and 7A,B). Cavities running along the threshold and vela are either completely absent or if visible, not as distinct as in the UVTT (Figs 4B,C, 5B and 6A,B). The door is hold at an acute angle of approximately 55°, except for *U. aureomaculata* and *U. flaccida* with ca. 40° door-threshold angles. Large amounts of mucilage, which mostly stick to the door edge and the threshold, are visible (Figs 3C,D, 4B,C, 5B, 6B and 7B). The trapdoor appears undulated in the set position, with the upper and lower parts being concave and the middle part with the trigger hairs being convex when seen from the outside. After triggering, the trigger hairs (Figs 3C–E, 4B,C, 5B, 6A,B and 7B,C) flip downwards. The trapdoor obtains a more or less overall concave curvature (*U. praelonga* duration: mean 15 ± 13.1 ms, range 6–46 ms, n = 7) and then swings open, with the trigger hairs still pointing downwards and towards the trap lumen (duration: mean 3.4 ± 0.5 ms, range 3–4 ms, n = 7). By reversal of the concave curvature, the door then re-closes (duration: mean 20.3 ± 7.5 ms, range 11–33 ms, n = 7) and the trigger hairs regain more or less their initial orientations. Patterns of cellular constrictions may be present on the inner trapdoor surface (Figs 3F, 4D, 6C and 7D).

### The *U. uniflora* trapdoor type (UUTT)

**Figure 8:**
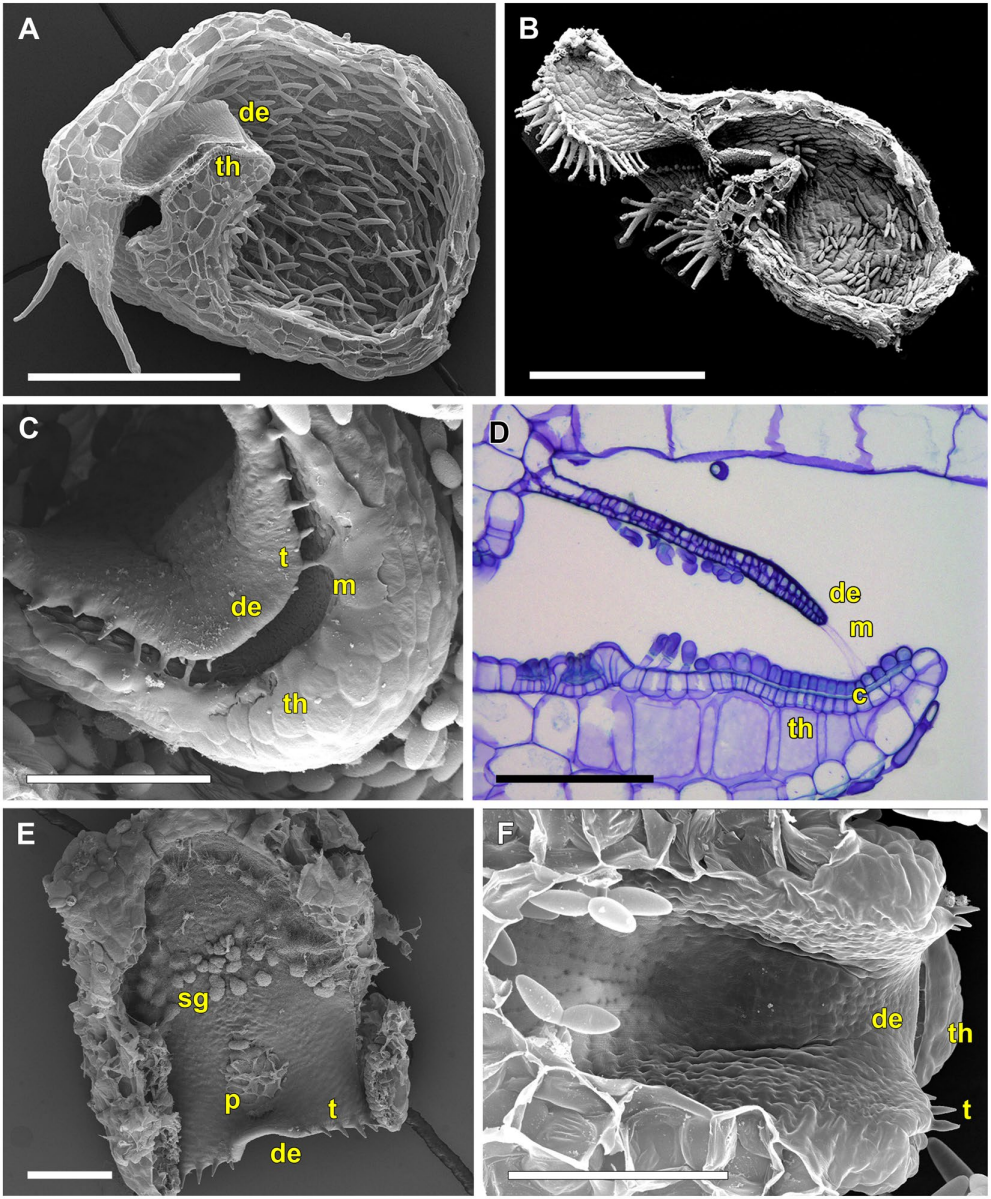
SEM and light microscopy (LM) images showing morphological trap characteristics of *U. uniflora* (representing the UUTT1), *U. menziesii* (representing the UUTT2), *U. warburgii* and *U. cornuta* (both representing the UUTT3). (**A**) Sagittal section of a trap of *U. menziesii* with the free door edge resting on the inner region of the threshold, reaching far into the trap lumen. The entrance is narrow and tubular, and covered by appendages on the outside. (**B**) Sagittal section of a trap of *U. warburgii*. (**C**) View on the inner door surface of *U. uniflora*. No concentric constrictions are visible. (**D**) View on the outer door surface of *U. uniflora*. Sessile glands in the upper part of the door as well as a pad of unknown function and teeth-like-structures are visible. (**E**) Detail of the inner door surface and the threshold with conspicuous teeth-like structures on the door edge as well as mucilage adhering to the teeth and the threshold. (**F**) LM sagittal thin-section (toluidine blue staining) of the trap entrance of *U. cornuta*. Perceptible are the cavity on the threshold and a thread of mucilage between the free door edge and the cavity. Scale bars A,B = 500 µm, C–F = 100 µm. Abbreviations: c = cavity, d = door, de = door edge, m = mucilage, p = pad, sg = sessile glands, t = teeth, th = threshold.

**Figure 9:**
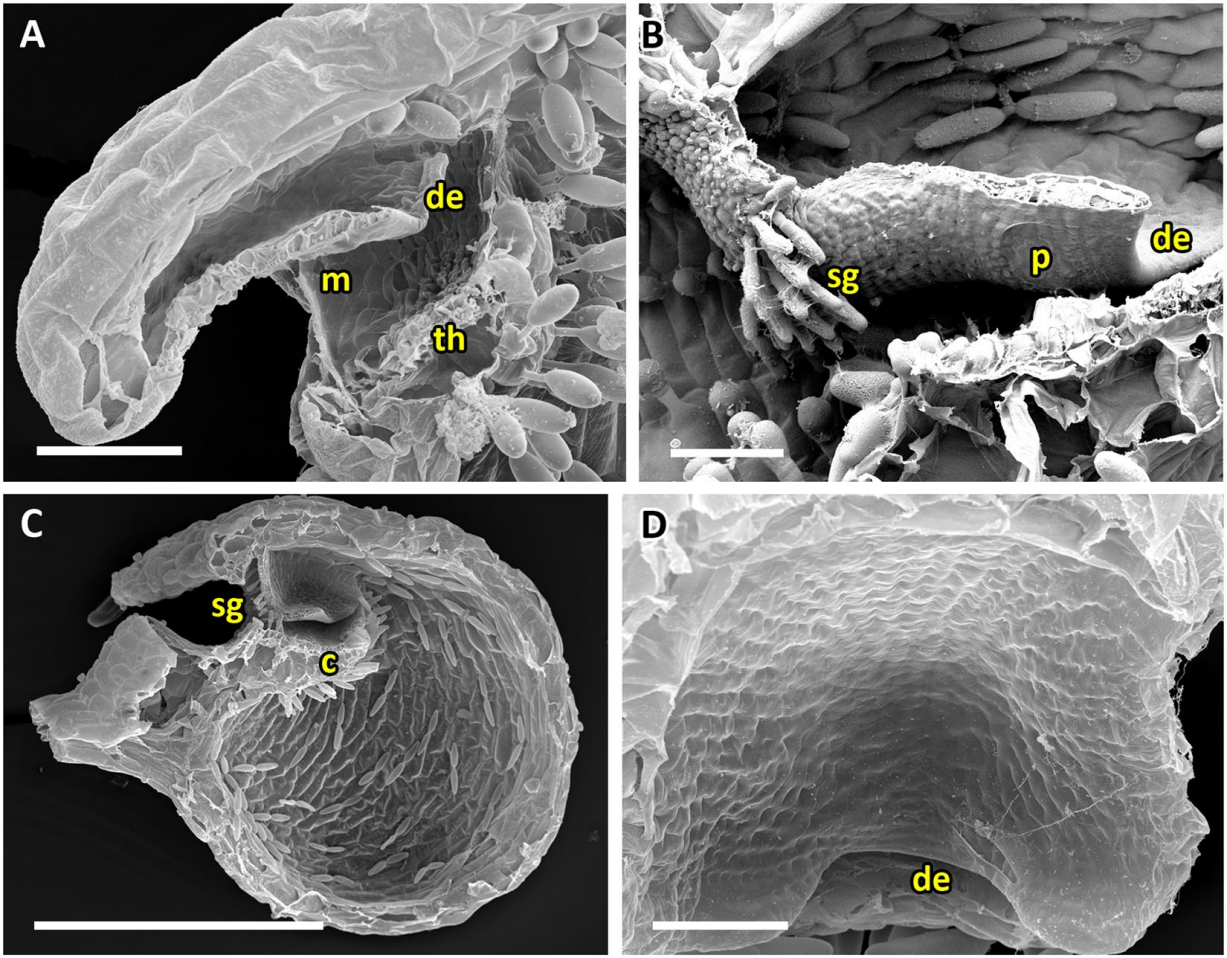
SEM and LM images of traps, trap entrances and trapdoors of *U. cornuta*, *U. warburgii* and *U. prehensilis* representing the UUTT3. (**A**) Sagittal section through the narrow, tubular trap entrance of *U. cornuta* (scale bar = 50 µm). (**B**) Sagittal section of a trap entrance of *U. warburgii* (scale bar = 50 µm). Note the free door edge, the fringe of long sessile glands presumably functioning as trigger hairs and the conspicuous pad on the door. (**C**) Sagittal section of a trap of *U. prehensilis* (scale bar = 500 µm). The trap entrance is tubular. On the trapdoor, sessile glands are observable and on the threshold a cavity. (**D**) View on the inner trapdoor surface. No pattern of concentric constrictions is visible (scale bar = 50 µm). Abbreviations: c = cavity, de = door edge, m = mucilage, p = pad, sg = sessile glands, th = threshold.

**Figure 10:**
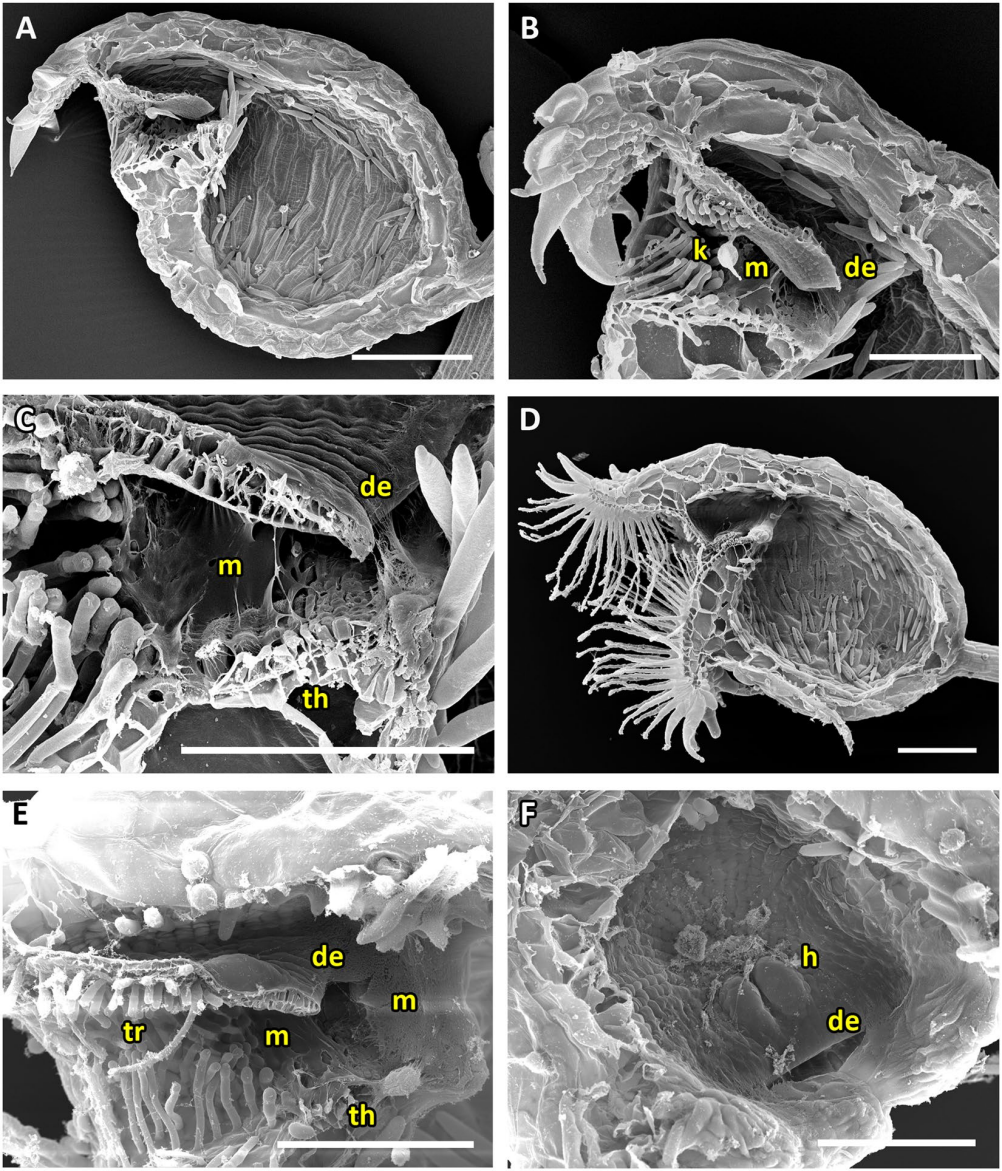
SEM pictures of traps, trap entrances and trapdoors of *U. welwitschii* and *U. livida*, representing the UUTT4. (**A**) Sagittal section through a trap of *U. welwitschii* (scale bar = 200 µm). Note the tubular trap entrance. (**B**) Sagittal section through a trap entrance of *U. welwitschii* (scale bar = 100 µm). Clearly visible are the free door edge, mucilage sticking to door and threshold and the noticeable, species-specific trigger hair, the so-called kriss-trichome. (**C**) Detail of mucilage sticking to the door of *U. welwitschii* (scale bar = 100 µm). (**D**) Sagittal section of a trap of *U. livida* (scale bar = 200 µm). Numerous appendages covering the tubular trap entrance can be seen. (**E**) Sagittal section through the trap entrance of *U. livida* (scale bar = 100 µm). A trigger hair protruding from the trapdoor is visible as well as mucilage adhering to the threshold and the free door edge. (**F**) View on the inner surface of *U. livida* with two humps visible on the trapdoor (scale bar = 100 µm). Abbreviations: de = door edge, h = hump, k = kriss-trichome, m = mucilage, th = threshold, tr = trigger hairs.

In traps of the UUTT, no door curvature inversion takes place prior to trap door opening (Movies S4–S7). The free door edge detaches from the threshold after triggering and then the door swings open. All traps of the UUTT possess tubular entrance regions (Figs 8A,B, 9A–C and 10A–D) and acute door-to-threshold angles of ca. 20–40°. Mucilage, which mostly sticks to the door edge and threshold, was noted in *U. uniflora*, *U. cornuta*, *U. welwitschii* and *U. livida* (Figs 8C,D, 9A,C and 10B,C,E). *U. warburgii*, which features a pad-like structure on the outer door surface (Fig. 9B), and *U. prehensilis*, which – as an exception of all UUTT species investigated – possesses a cavity (Fig. 9C), have no mucilage according to our scanning electron microscopy (SEM) survey. Vela and cavities on the threshold are not (*U. uniflora*, *U. dichotoma* and *U. menziesii*), or not distinctly visible (Fig. 8D).

This type can be subdivided into four subtypes. The UUTT1 is found in *U. dichotoma* and *U. uniflora* (Movie S4), both perennials from *U*. sect. *Pleiochasia*. The trapdoor has no trigger hairs but possesses conspicuous sessile or stalked glands (Fig. 8E). In the set position, the door appears as strongly undulated. Its upper part is markedly convex, whereas the middle and lower parts together are displaced backwards (towards the trap lumen). This compound middle and lower part possesses a posture similar to the overall door posture described above for the UPTT. After triggering, the lower free edge of the trapdoor detaches from the threshold in a comparably slow manner (duration: *U. uniflora* mean 632.1 ± 823.5 ms, range 19–2564 ms, n = 8; *U. dichotoma* mean 11 ± 5.9 ms, range 5–19 ms, n = 3). The door then swings open without notably changing its initial curvature (duration: *U. uniflora* mean 5.3 ± 1.28 ms, range 4–8 ms, n = 8; *U. dichotoma* mean 4 ± 1.4 ms, range 2–5 ms, n = 3). During re-closure, the individual curvature of the different trapdoor regions decreases in magnitude, leaving only a slight S-shape of the door (duration: *U. uniflora* mean 238.5 ± 405.8 ms, range 11–1240 ms, n = 8; *U. dichotoma* mean 42.3 ± 25.8 ms, range 12–75 ms, n = 3). By this closure motion, the door does not yet regain the initial posture as it was observed in the respective set position. Patters of concentric cellular constrictions are not visible (Fig. 8F). The lower outer door surface *U. dichotoma* and *U. uniflora* show conspicuous pad-like structures (Fig. 8E). On the lateral door edges of *U. uniflora,* teeth-like structures exist (Fig. 8C,E,F).

**Figure 11:**
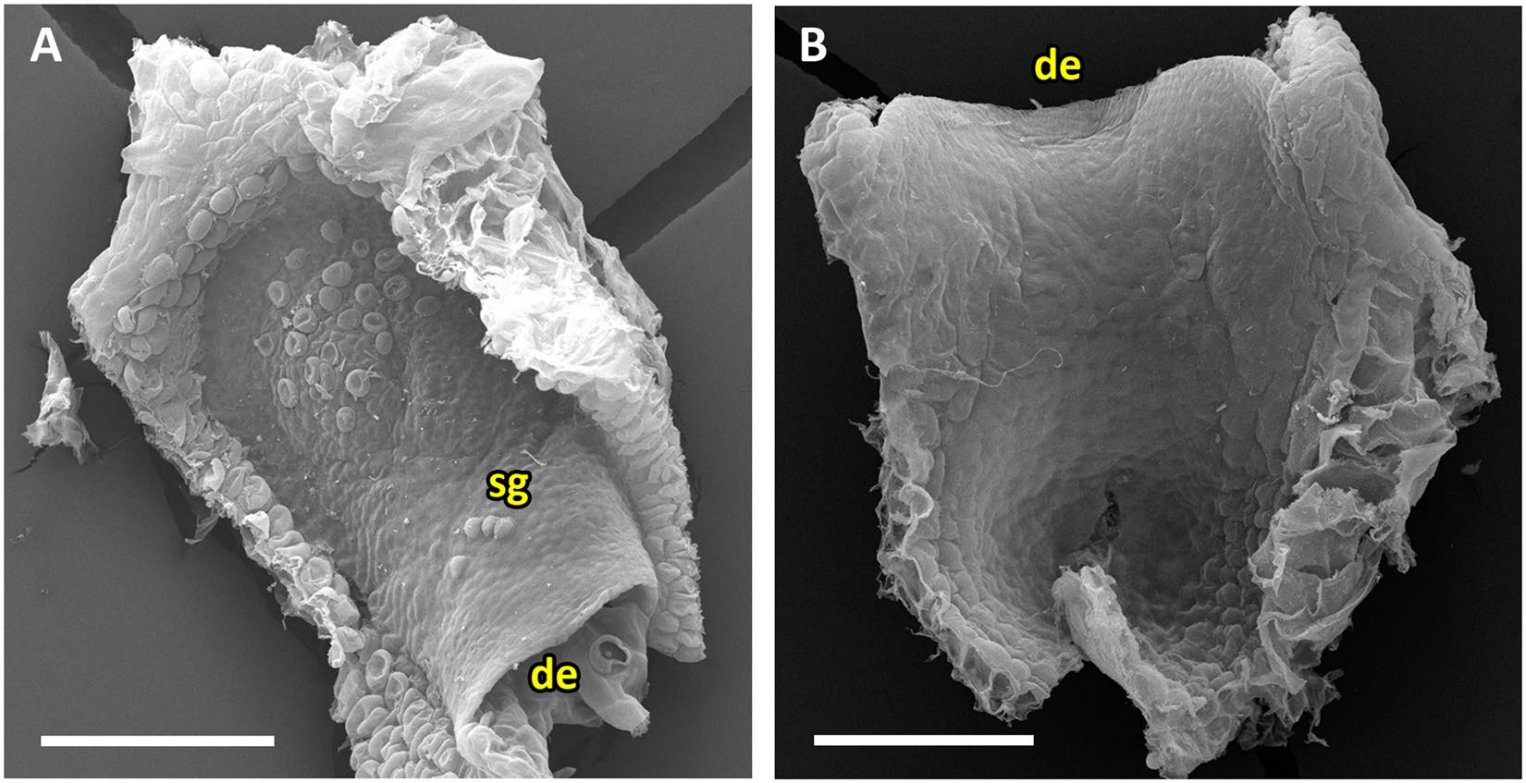
Trap door of *U. menziesii* (UUTT2). (**A**) View on outer door surface. (**B**) View on the inner door surface. The door maintains a S-shaped curvature though freed from the trap entrance. Abbreviations de = door edge, sg = sessile glands. Scale = 100 µm.

The second subtype (UUTT2) is found in the seasonally growing geophyte *U. menziesii* of *U*. sect. *Pleiochasia* (Movie S5). The door has no trigger hairs but possesses conspicuous sessile or stalked glands (Fig. 11A). No concentric cellular constrictions on the inner door surface are visible. The door is oblong and, when freed from surrounding trap entrance tissue, maintains a sigmoid curvature (Fig. 11B). It is markedly concave in its entirety when the trap is ready to fire. After triggering, the free edge detaches from the inner region (duration: mean 6.5 ± 2.0 ms, range 4–9 ms, n = 6). The door then opens and obtains a shape which resembles a mirror-inverted UPTT set position (duration: mean 3.2 ± 1.1 ms, range 2–5 ms, n = 6). The door re-closes by changing to a slight convex curvature (duration: mean 20.2 ± 26.5 ms, range 3–75 ms, n = 6), hereby blocking the tubular trap entrance with its middle part. By this closure motion, the doors and trigger hairs do not yet regain the initial postures as in the respective set positions.

The UUTT3 is found in *U. cornuta* (*U*. sect. *Stomoisia*), *U. prehensilis* (*U*. sect. *Oligocista*) and *U. warburgii* (*U*. sect. *Nigrescentes*), and presumably *U. rostrata* (*U*. sect. *Aranella*) (Movie S6). Here, the door possesses conspicuous sessile or stalked glands instead of trigger hairs (Fig. 9B,C). In set position, the door is only slightly undulated and has the shape of a mirror inverted ‘S’. After triggering (like in the UUTT1), detachment from the threshold takes place (duration: *U. cornuta* mean 4.7 ± 0.5 ms, range 4–5 ms, n = 4; *U. prehensilis* mean 11.4 ± 7.2 ms, range 1–29 ms, n = 10; *U. warburgii* mean 24.2 ± 26.9 ms, range 5–74 ms, n = 6). Detachment is followed by opening (duration: *U. cornuta* mean 4 ± 2.5 ms, range 1–8 ms, n = 4; *U. prehensilis* mean 4.1 ± 1.0 ms, range 3–6 ms, n = 10; *U. warburgii* mean 3 ± 1.5 ms, range 3–5 ms, n = 6) during which the door gains a slightly concave curvature that is reversed during re-closure (duration: *U. cornuta* mean 18.3 ± 7.9 ms, range 9–27 ms, n = 4; *U. prehensilis* mean 13.4 ± 74.0 ms, range 8–21 ms, n = 10; *U. warburgii* mean 12 ± 11.4 ms, range 2–36 ms, n = 6). Hereby, the trap entrance becomes closed by the free edge of the trap door. Trap door and trigger hairs do not yet regain the initial posture as in the respective set position by this closure motion. No cellular constriction pattern can be detected (Fig. 9D). On the lower outer door surface of *U. warburgii*, like in *U. dichotoma* and *U. uniflora* (both UUTT1), conspicuous pad-like structures can be seen which remind of crumpled cushions (Figs 8E, 9B).

The UUTT4 is found in *U. livida* and *U. welwitschii* of *U*. sect. *Calpidisca* (Movie S7). In contrast to the otherwise structurally similar UUTT3, the door possesses trigger hairs (Fig. 10B,E). The door movements are similar to those described for the UUTT3. Circular cellular constriction patterns are not present. On the inner door surface of *U. livida* two peculiar bumps can be noted (Fig. 10F).

Since no information on the presence of trigger hairs could be gained by SEM for *U. rostrata*, it is not clear if this species belongs to UUTT3 or UUTT4.

### The *U. multifida* trap type

Whereas in all other investigated species trap firing and trapdoor movement could be triggered without any problem, we did not observe any trap action in *U. multifida* of *U*. sect. *Polypompholyx* (see also results on the SFs) and hence, no trapdoor movement type can be assigned. Therefore, we propose establishing the passive *U. multifida* trap type.

**Figure 12:**
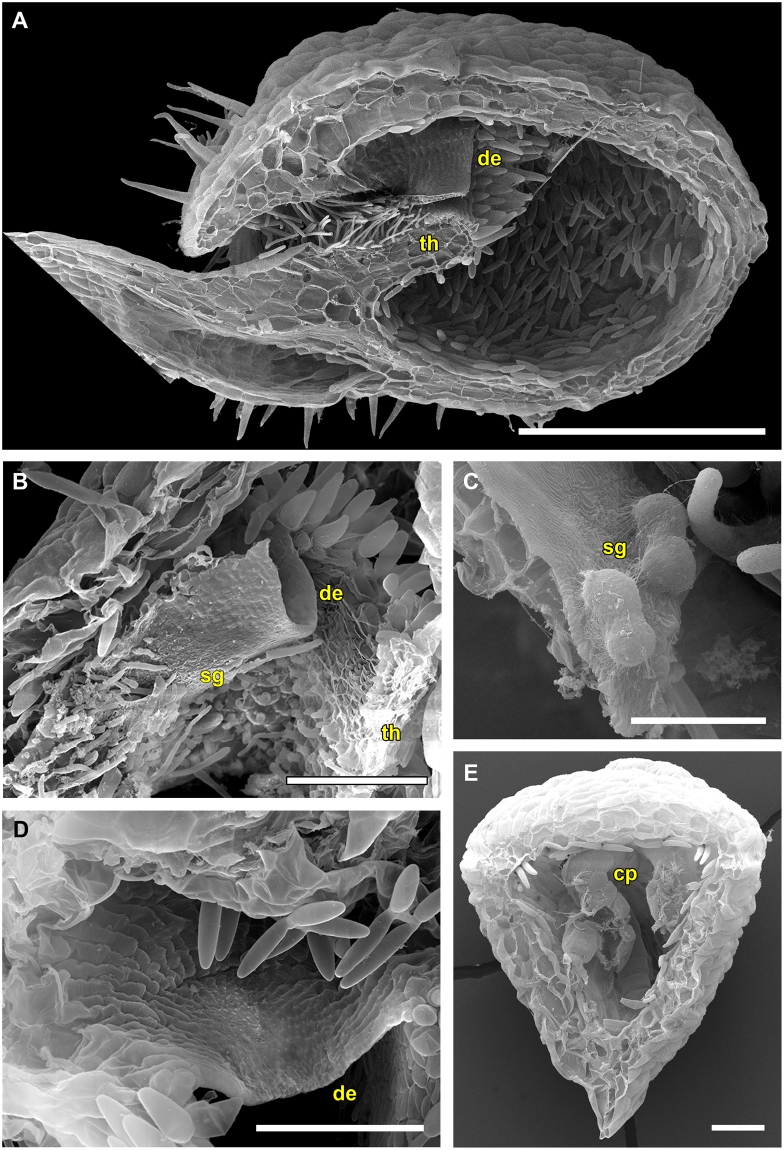
SEM images showing morphological trap characteristics of *U. multifida*. (**A**) Sagittal section through a trap (scale bar = 500 µm). The long, tubular and narrow trap entrance is densely covered with glands pointing towards the trapdoor. The free door edge and the threshold are visible. (**B**) Sagittal section through the trap entrance with intact trapdoor (scale bar = 100 µm). Note the small sessile glandular structures on the trapdoor. (**C**) Detail view of the sessile glandular structures (scale bar = 20 µm). (**D**) View on the inner surface of the trapdoor (scale bar = 100 µm). Concentric cellular constrictions could not be detected. (**E**) Posterior part of a bladder filled with caught copepods (scale bar = 100 µm). Note the characteristic triangular shape of the *U. multifida* bladder and its thick and putatively stiff trap walls. Abbreviations: cp = copepod, de = door edge, sg = sessile glands, th = threshold.

The tubular trap entrance is densely covered with long glands pointing towards the trapdoor and reminds of a fyke (Fig. 12A). It is further obscured by the stalk and appendages. Sessile glandular structures are present on the trapdoor (Fig. 12B,C). The door is morphologically not conspicuously different from that of *U. menziesii* (Figs 8A, 11A and 12A,B,D). Concentric cellular constrictions are not present on its inner surface (Fig. 12D). The lateral, 3-cell-layered trap walls are ca. 90 µm thick (Fig. 12E).

### Suction dynamics

**Figure 13:**
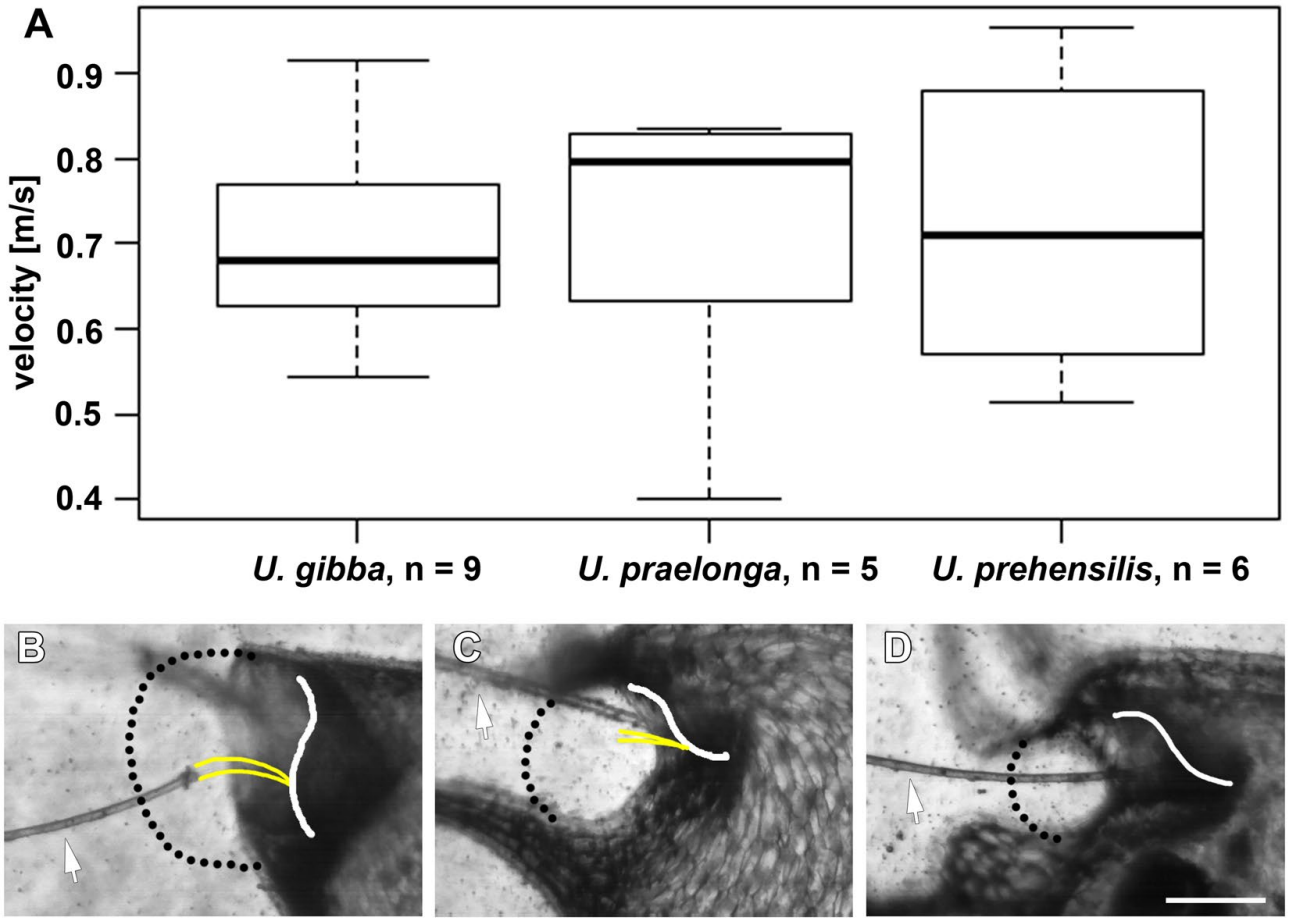
Fluid velocities and aspiration zones in three *Utricularia* species representing the three main trapdoor movement types. (**A**) The top fluid speeds among the three tested species do not differ statistically (Kruskal-Wallis test, χ^2^ (2) = 0.24698, *p* = 0.884). (**B–D**) Lateral views on trap entrances showing the aspiration zones (dotted lines). The set positions of the doors (white lines) with trigger hairs (where present, yellow lines) are indicated. Also visible are the hair used for manually triggering the traps (arrows), as well as a multitude of tracer particles used to study the flow of water during suction. (**B**) Aquatic *U. gibba* (UVTT). (**C**) Terrestrial *U. praelonga* (UPTT). (**D**) Terrestrial *U. prehensilis* (UUTT3). Scale bar (applies for **B**–**D**) = 100 µm.

The comparison of traps with similar size (length ca. 1 mm) from *U. prehensilis* (UUTT3), *U. praelonga* (UPTT) and *U. gibba* (UVTT) does neither reveal significant differences in top fluid speed (Kruskal-Wallis test,χ^2^ (2) = 0.24698, *p* = 0.884) nor in acceleration during the suction process (*U. prehensilis* top fluid speed 0.9 m/s and maximal acceleration 679 *g, U. praelonga* 0.8 m/s and 648 *g, U. gibba* 0.9 m/s and 836 *g*) (Movies S1,S8,S9). The suction process in *U. gibba* lasts between 1.5–3.1 ms, in *U. praelonga* between 1.4–1.7 ms and in *U. prehensilis* between 0.9–1.3 ms (Fig. 13). Reynold’s numbers are according to the wide entrance higher in *U. gibba* (RE = 216) than in the narrow entrances of *U. prehensilis* (RE = 100) and *U. praelonga* (RE = 99). Additionally, the aspiration zone measured for *U. gibba* (19,300 ± 3,600 µm²) is approximately 1.5 times wider than in *U. prehensilis* (11,100 ± 1,600 µm²) and in *U. praelonga* (12,800 ± 1,900 µm²) (Fig. 13).

### Spontaneous firings (SFs)

In all species investigated, with the exception of *U. multifida*, SFs with the different modes (metronomic, random, and bursts) were recorded (Table 1). *U. menziesii* spontaneously fired once during high-speed recording experiments. Some species (*U. calycifida*, *U. dichotoma, U. longifolia*, *U. welwitschii*) also showed different firing behaviours among traps on the same stolon. Although traps of *U. multifida* were intensively studied with identical methods as applied for the other species regarding the occurrence of spontaneous firings (see Discussion), neither firing nor trap deflation processes could be observed. The plants showed signs of growth and organ reorientation during these studies, indicating that they were alive and physiologically active.

### Phylogenetic tree and ancestral trap character states

**Figure 14:**
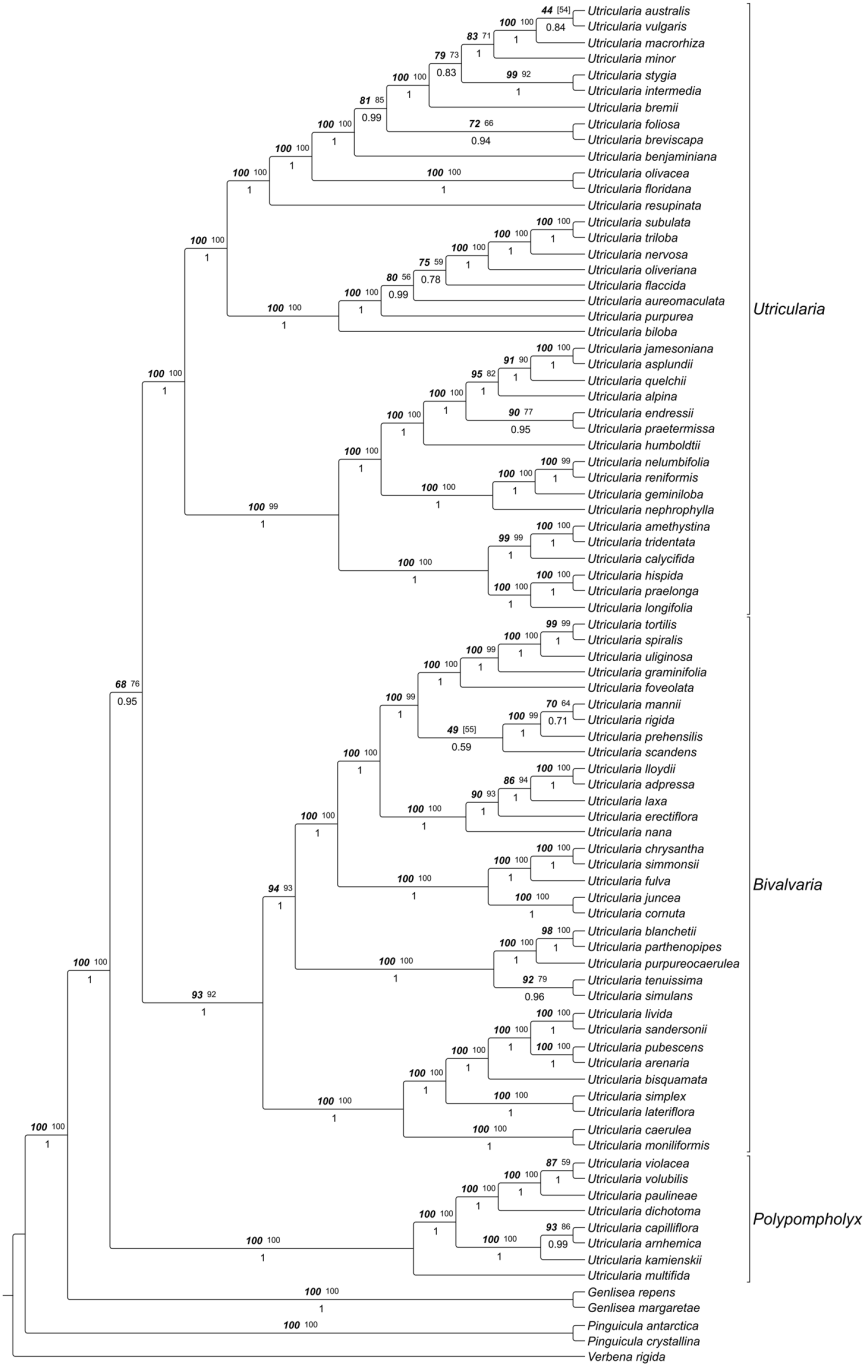
Phylogeny of *Utricularia* as inferred with Maximum Likelihood, Maximum Parsimony and Bayesian Inference of *trnK/matK* and *trnL-F* sequences. Tree topology was taken from the ML analysis, numbers above branches indicate ML bootstrap percentages (bold italics; right), and MP bootstrap percentages (plain, left), numbers below branches are posterior probabilities from Bayesian Inference.

The molecular dataset combined of *trnK/matK* and *trnLF* sequences for 84 taxa yielded an alignment of 5381 characters, 907 of which were excluded from subsequent analysis because of uncertain homology. Consensus trees from parsimony analyses were well resolved and supported. The Maximum Parsimony (MP) trees from substitutions only were 7534 steps long (Consistency Index, CI 0.490; Retention Index, RI 0.709; Rescaled Consistency Index, RC 0.348), those based on substitutions and indel characters combined had a length of 8209 steps (CI 0.513, RI 0.710, RC 0.487). The Maximum likelihood (ML) analysis yielded one optimal tree with a likelihood score of -46985.092291. The tree topology from this search is shown in Fig. 14, collapsing nodes supported by less than 50% in at least one of the tree methodological approaches. Trees from Bayesian inference (BI) and ML generally showed slightly higher resolution and statistical support than trees from MP searches.

**Figure 15:**
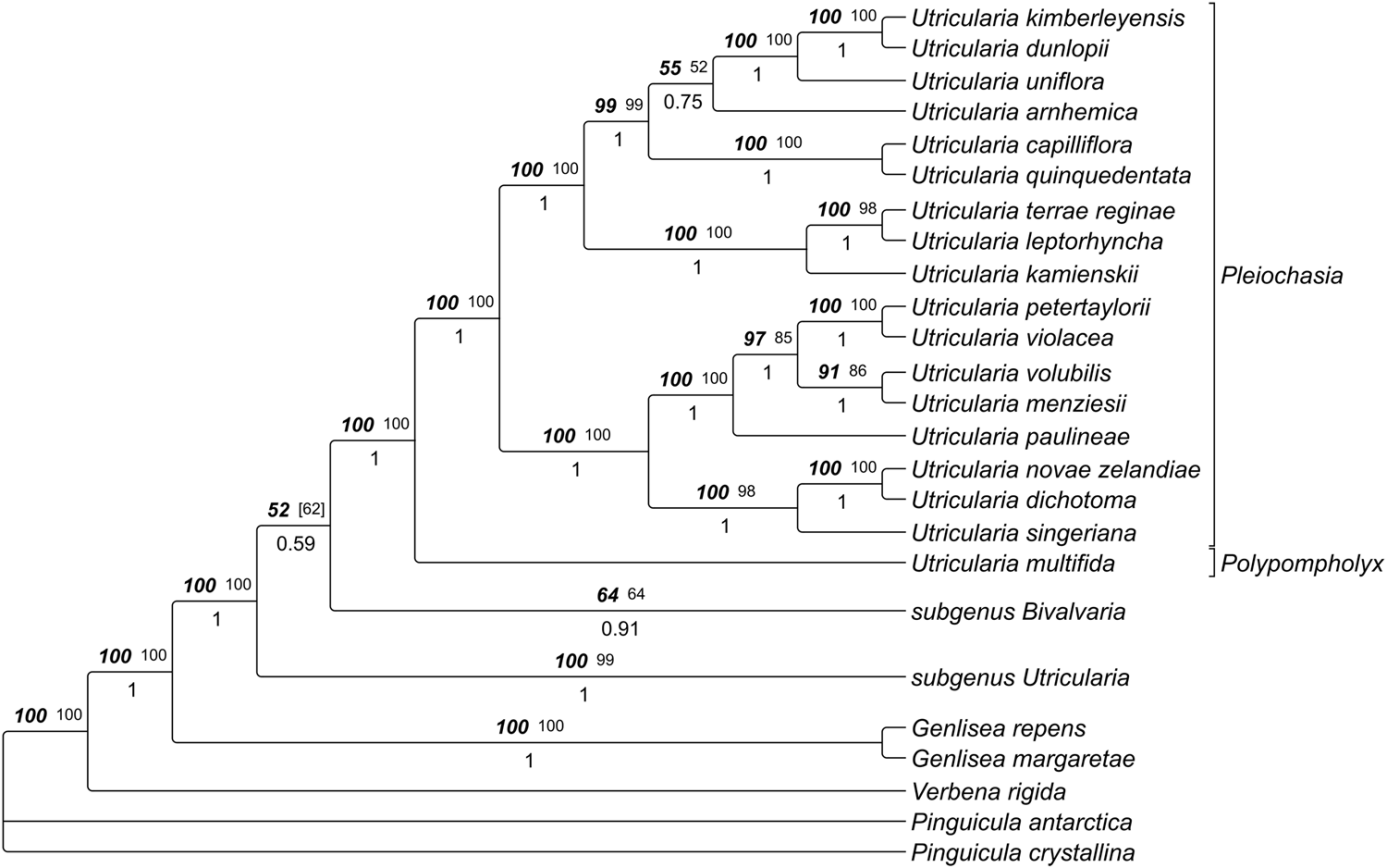
*U*. subgenus *Polypompholyx*, as inferred with Maximum Likelihood, Maximum Parsimony and Bayesian Inference of *trnK/matK* sequences. Tree topology was taken from the ML analysis, numbers above branches indicate ML bootstrap percentages (bold italics; right), and MP bootstrap percentages (plain, left), numbers below branches are posterior probabilities from Bayesian Inference. Classification according to ref.^5^, as modified by ref.^9^, is indicated to the right of the tree.

**Figure 16:**
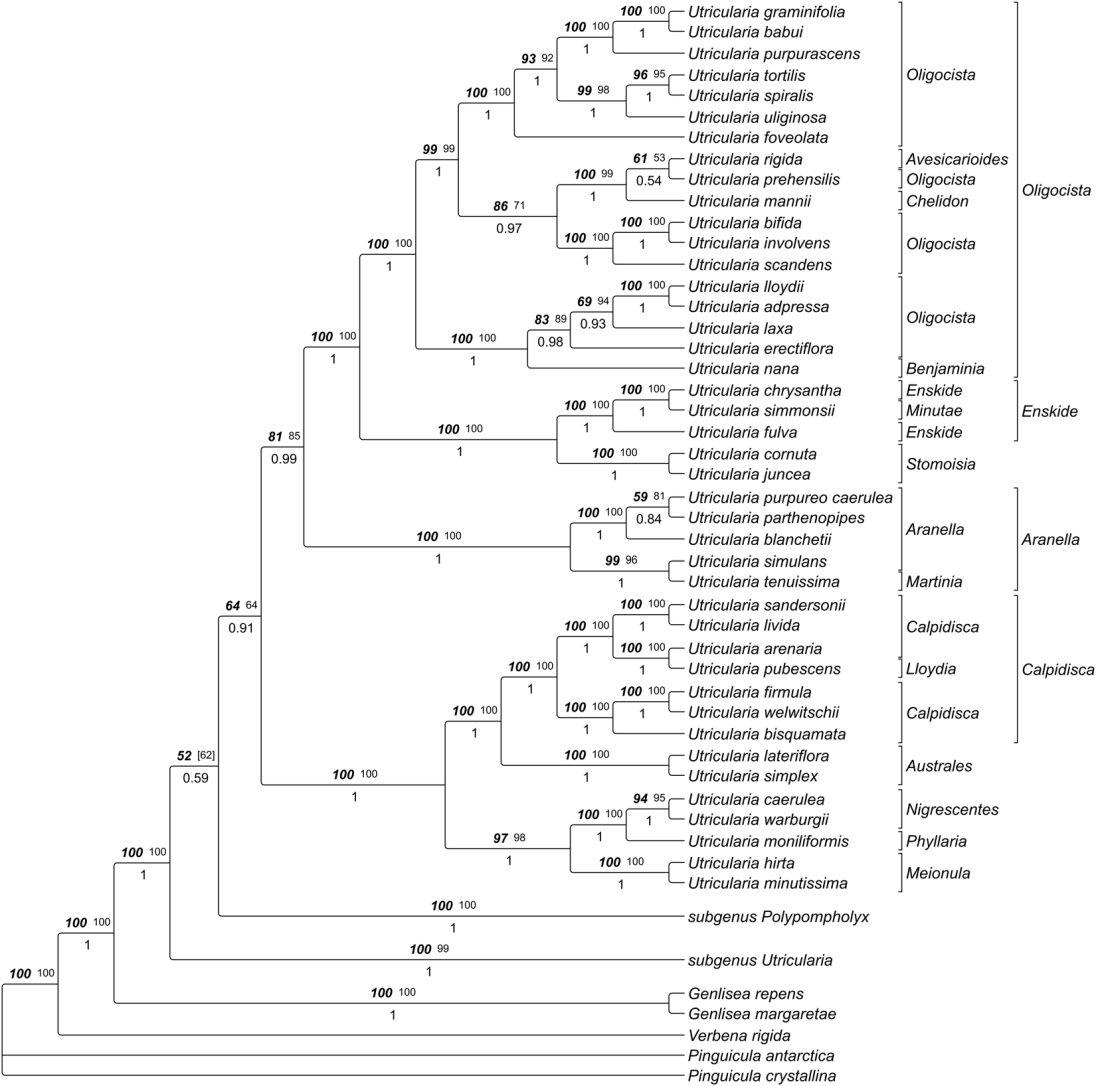
Phylogeny of *U*. subgenus *Bivalvaria* as inferred with Maximum Likelihood, Maximum Parsimony and Bayesian Inference of *trnK/matK* sequences. Tree topology was taken from the ML analysis, numbers above branches indicate ML bootstrap percentages (bold italics; right), and MP bootstrap percentages (plain, left), numbers below branches are posterior probabilities from Bayesian Inference. Classification according to ref.^5^, as modified by ref.^9^, is indicated to the right of the tree.

**Figure 17:**
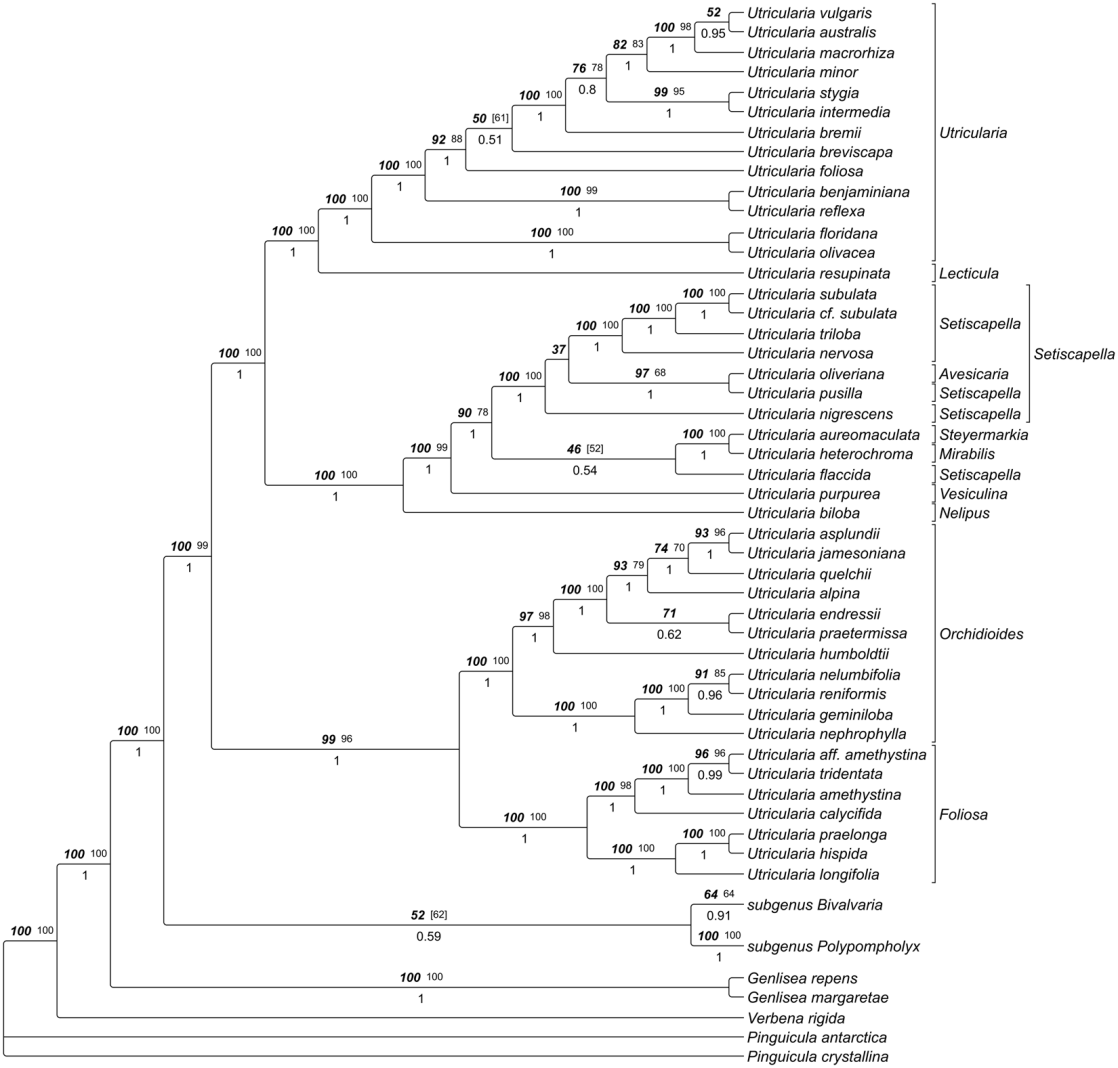
*U*. subgenus *Utricularia*, as inferred with Maximum Likelihood, Maximum Parsimony and Bayesian Inference of *trnK/matK* sequences. Tree topology was taken from the ML analysis, numbers above branches indicate ML bootstrap percentages (bold italics; right), and MP bootstrap percentages (plain, left), numbers below branches are posterior probabilities from Bayesian Inference. Classification according to ref.^5^, as modified by ref.^9^, is indicated to the right of the tree.

The *trnK/matK* dataset (110 spp.) yielded an alignment of 3634 characters of which 593 were excluded from subsequent analysis because of uncertain homology. Consensus trees from parsimony analyses were well resolved and supported. The MP trees from substitutions only were 6554 steps long (CI 0.448, RI 0.731, RC 0.327), those based on substitution and indel characters had a length of 6853 steps (CI 0.463, RI 0.736, RC 0.341). The likelihood analysis yielded one optimal tree with a likelihood score of -40217.451022. The tree topologies from this search are shown in Figs 15–17, collapsing nodes supported by less than 50% in at least one of the tree methodological approaches.

## Discussion

Based on our comparative biomechanical and functional-morphological studies, we propose the distinction between *Utricularia* trap and trapdoor movement types based on differences in functional principle, entrance morphology (short vs. tubular), angles between the doors and the thresholds (obtuse or acute), and trapdoor motion (curvature inversion prior to opening, or no curvature inversion and comparably slow door edge detachment from threshold) (Fig. 1). According to our analyses, *U. multifida* traps do not function via suction. In order to understand these trap and trapdoor movement types in an evolutionary context, we employed a refined phylogenetic reconstruction of the genus based on new molecular data. Our analysis recovered the same three major clades that were obtained in previous studies (Fig. 14)^6,9^. We additionally find the *U*. sect. *Australes*, *Chelidon*, *Enskide* (including *Minutae*, see ref.^22^), *Meionula*, *Nigrescentes*, and *Phyllaria* in *U*. subg. *Bivalvaria* (sensu ref.^9^). *Utricularia* sect. *Avesicaria*, *Lecticula*, *Mirabiles*, and *Steyermarkia* are found in a clade together with taxa assigned to *U*. subg. *Utricularia*. The major discrepancies between tree topologies from *trnK/matK* ^9^ and those from combined *trnL-F* and *rps16* data^6^ are the relationships between these three subgenera. The three major phylogenetic clades correspond well with the observed functional, kinematical and morphological characteristics, as discussed in the following.

### Utricularia subgenus Polypompholyx

Within the phylogenetically early-branching *U*. subg. *Polypompholyx*, *U. multifida* from *U*. sect. *Polypompholyx* is sister to a clade containing all species from *U*. sect. *Pleiochasia* (Fig. 15). This section clearly appears as monophyletic. A recent study using the *rps16* region as a phylogenetic tool did not find the monophyly of *U*. sect. *Pleiochasia*, but instead found two clades consisting of taxa from that section and a clade including taxa from *U*. sect. *Polypompholyx* unresolved^22^. In our phylogenetic study, the species of *U*. sect. *Pleiochasia* form two well-supported clades. The first clade comprises *U. dichotoma* (UUTT1)*, U. menziesii* (UUTT2), *U. novae-zelandiae, U. paulineae, U. petertaylorii, U. singeriana, U. violacea* and *U. volubilis*, and corresponds to clade 1 of ref.^22^. In the second clade, *U. arnhemica, U. capilliflora, U. dunlopii, U. kamienskii, U. kimberleyensis, U. leptorhyncha, U. quinquedentata, U. terrae-reginae*, and *U. uniflora* (UUTT1) are found. This group conforms to clade 2 of ref.^22^. Taxa from clade 1 are found mostly in south-west to south-east regions of Australia. Those from clade 2 are distributed in the north of the Tropic of Capricorn^22^. However, the authors mention that this geographic division is not very sharp.

The monophyly of *U*. sect. *Polypompholyx* was shown recently, but the monotypic *U*. sect. *Tridentaria*, which might have close affinities to *U*. sect. *Polypompholyx*, has not been sampled to date. However, biomechanical and functional-morphological studies of *U. westonii* from that section would be of interest in the light of phylogeny and trap function, as this species bears comparatively large and stiff traps of triangular shape^5^.

*U. multifida* (*U*. sect. *Polypompholyx)* most probably possesses an exceptional passive trap type. According to our observations, the traps do not capture prey by suction but work in a passive manner similar to closely related *Genlisea* corkscrew plants (eel- or lobster-traps)^23,24^. The darkish traps of *U. multifida* are probably highly attractive for shelter-seeking prey but it could also be speculated that other means of attraction (mucilage, chemical substances) play a role. However, this conclusion has to be regarded as preliminary (cf. refs^15,22^), because we cannot entirely rule out that suction would occur at some developmental stage(s) of the trap (e.g. only in young traps). We investigated traps in March 2015 (two plants with four traps) and April 2016 (four plants with eight traps) in respect to their suction action and trapdoor movements. Traps were repeatedly stimulated with a fine human hair and a thicker nylon thread in intervals lasting several hours for two days in 2015, and for three days in 2016. The experiments were otherwise identical with all other experiments on *Utricularia* traps (procedures, equipment, environmental conditions). Furthermore, A.F. tested the traps in the field and on cultivated specimen numerous times by touching the trapdoors with a human hair or thin grass blades, but active suction (indicated by shape change of the trap walls) was never observed. The trap inactivity could theoretically be explained by (1) unfavorable environmental conditions during the experiments (although the plants were undoubtedly alive and physiologically active, see Results), and all other species investigated were successfully tested for suction under the same conditions), (2) traps were in a wrong developmental stage (which is highly doubtful due to the ephemeral nature of *U. multifida* and as the traps investigated were fully developed, plus they had been studied twice independently in two subsequent years), and (3) traps suck only once (which was not observed) and are not capable of water pumping/resetting. As the authors of ref.^25^ discuss for species of the closely related *U*. section *Pleiochasia*, traps with multi-layered (stiff) walls exhibit a lag-period of water pumping after trap firing. It is conceivable that *U. multifida*, with its peculiar trap body and thick walls, either has an extraordinary long lag-period or even no resetting mechanism. Glands are found at the inner threshold surface (Fig. 12A) where the glands for water pumping are typically situated (as known from aquatic species). However, these glands may differ in morphology (e.g., number of ‘arms’) and hence may not be certainly identifiable^5^. Apart from larger copepods (Fig. 12E), we found several small nematodes inside the traps of cultivated *U. multifida* that could be considered as potentially being too weak to pull open the trapdoor but are perfectly built to squeeze through the tightest trap entrances.

*U. multifida* is the only species in which no SFs were observed. This otherwise general trap behaviour leads to accumulation of biomass, which adds to *Utricularia* nourishment^21^.

### *Utricularia* subgenus *Bivalvaria*

With respect to those *Utricularia* sections for which trap data are reported, the most basal split in *U*. subgenus *Bivalvaria* (Fig. 16) suggests close affinities between *U*. sect. *Calpidisca* and *Nigrescente*s on the one hand, and *Aranella*, *Stomoisia*, and *Oligocista* on the other.

*Calpidisca* in its current circumscription is found to be paraphyletic, since there is maximal support for a group including all members from *U*. sect. *Calpidisca* plus *U. pubescens* from the monotypic *U*. sect. *Lloydia*. In earlier phylogenetic studies^6,9^, this paraphyly of *U*. sect. *Calpidisca* was not observed, probably due to the sparser taxon sampling.

Ref.^5^ suggested *U. pubescens* (monotypic *U*. sect. *Lloydia*) to be closely related to *Calpidisca*, but established a section of its own for this species based on some morphological differences in leaves, seeds, bracts and bracteoles. The author argued that *U*. sect. *Calpidisca* might be divided into two groups based on two different types of traps. The first group has complex trapdoor hairs, called ‘kriss trichomes’^15^ (represented by the UUTT4 and found in *U. welwitschii*). The other group has a pair of simple bristles on the trap door, as found in *U. livida* (represented in the UUTT3), *U. arenaria*, and *U. sandersonii* ^15^. Taylor^5^ rejected a subdivision of the section based on this character because there are no further differences supporting this. However, taxa from these two groups based on the trapdoor types (UUTT3 and UUTT4, see Fig. 1) do form well supported clades in our trees (Fig. 16). *U. pubescens* has traps very similar to the latter group, and is found in a clade together with them.

Members of *U*. sect. *Nigrescentes* resolve as sister to *U. moniliformis* (*U*. sect. *Phyllaria*). Taylor^5^ already suggested stronger affinities between those sections based on placenta morphology. *U*. sect. *Meionula* in turn is sister to the *Nigrescentes*/*Phyllaria* clade.

Among the clade comprising the remaining sections of *U*. subg. *Bivalvaria*, a group including *U*. sect. *Aranella* and *U. tenuissima* (monotypic *U*. sect. *Martinia*, which might need to be included within *U*. sect. *Aranella*) branches first. *U*. sect. *Stomoisia* that is inferred as monophyletic occurs in North and Central America and is sister to a strictly Australian group including paraphyletic *U*. sect. *Enskide*, with the minute species *U. simmonsii* from section *Minutae* nested inside.

Members of a clearly non-monophyletic *U*. sect. *Oligocista* are found scattered across a clade that also includes *U*. sect. *Avesicarioides*, *Chelidon*, and *Benjaminia*. Two groups become apparent. The first comprises South and Central American species of *U*. section *Oligocista* plus *Benjaminia*. Taylor^5^ already noted affinities between both, but assigned more weight to trap entrance appendages as distinguishing character. A second group includes the remaining species from *Oligocista* plus the epiphytic monotypic *U*. sect. *Chelidon* and the rheophytic *U*. sect. *Avesicarioides*. All species occur in the Old World tropics.

In *U*. subg. *Bivalvaria*, represented by the UUTT3 and UUTT4, species possess traps with a tubular trap entrance and obtuse-angled door that slowly detaches from the threshold after triggering. The structural features of cavities, vela and concentric constrictions are missing. Generally, this clade comprises exclusively non-aquatic species, however, only a limited number of species (not representing all sections of *U*. subg. *Bivalvaria*) has been studied so far.

Nonetheless, the UUTT in general is not a synapomorphy for members of *U*. subg. *Bivalvaria*, but is also found in the ‘derived’ members (i.e. *U*. sect. *Pleiochasia*) of *U*. subg. *Polypompholyx* (which is common sister to subgenera *Bivalvaria* and *Utricularia*), yet here established as subtypes UUTT1 and UUTT2. Only in the early-branching *U. multifida* (*U*. sect. *Polypompholyx*) and the unique geophyte *U. menziesii* (*U*. sect. *Pleiochasia*), different trap and trapdoor types have been observed.

### *Utricularia* subgenus *Utricularia*

The aquatic species investigated here are all from the clearly monophyletic *U*. subg. *Utricularia* (sensu ref.^9^) (including UVTT species known from earlier studies: *U. australis*, *U. inflata*, *U. vulgaris* ^17^) which is composed of two major clades (Fig. 17). The first includes *U*. sect. *Foliosa* and *Orchidioides*, both represented in our trap data sampling and by all non-aquatic species of *U*. subg. *Utricularia* that belong to the UPTT, with traps possessing tubular trap entrances and acute-angled doors that perform curvature inversions before opening. The second clade comprises several sections, out of which *U*. sect. *Utricularia*, *Setiscapella*, *Lecticula*, and *Steyermarkia* have been included in our trap analyses. Within the latter group, *U*. sect. *Lecticula* (two species only, both affixed aquatics, represented by the UVTT2) is sister to *U*. sect. *Utricularia* (represented by the UVTT1). Taylor^5^ also described affinities between these two, citing trap morphology, seeds and pollen as evidence. However, *U. resupinata* and *U. spruceana* are placed in their own section based on tubular bracts that are unique in the genus. Following the classification of Taylor^5^, all species of *U*. sect. *Lecticula* are affixed aquatics, while *U*. sect. *Utricularia* has both affixed and freely suspended aquatics. That author argued that the affixed aquatic habit is an intermediate step linking a terrestrial life form to a freely suspended one. Indeed, our results show the affixed *U*. sect. *Lecticula* as sister to *U*. sect. *Utricularia* (Fig. 17), which is further supported by the trapdoor subtypes with the different trigger hair movements. On the other hand, affixed aquatics appear somewhat randomly distributed inside *U*. sect. *Utricularia*, suggesting that freely suspended aquatics may have evolved multiple times from affixed ones. It may well be that the differentiation between affixed and suspended aquatics should be reevaluated. Note, however, that we only included 13 species out of 34 from *U*. sect. *Utricularia* ^5^ in the present study, so more work is needed before a clear picture of evolution of live forms in that section can emerge.

### Ecological and evolutionary implications of the different trap and trapdoor movement types

**Figure 18:**
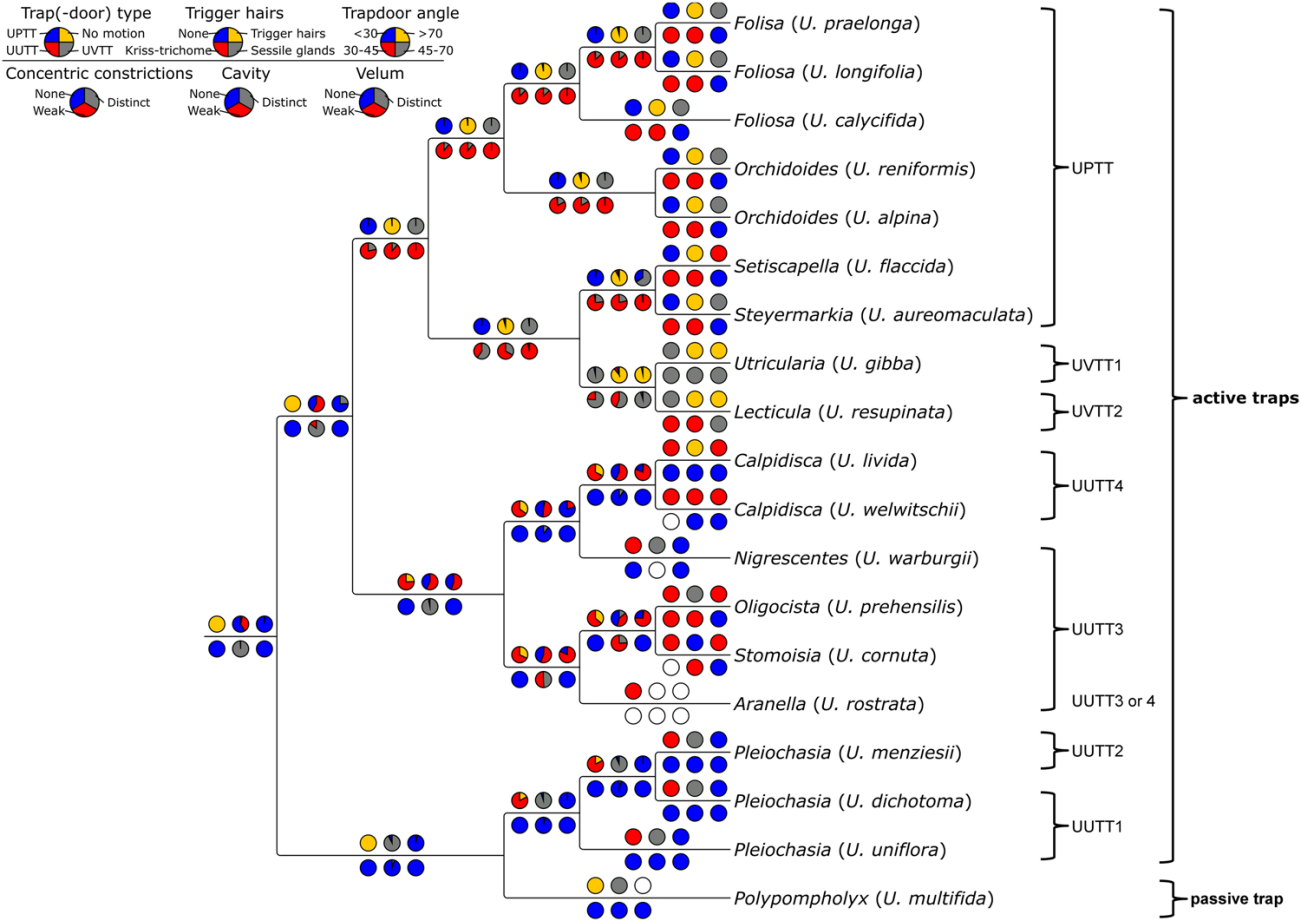
Relative state probabilities of trap characters at ancestral nodes. Positions of characters on branches (three above branches, three below branches) and colours of individual character states in the pie chart labels as indicated in the legend (top left). Full circle (all possible states; probability = 1.0, 100%) is divided into sectors (representing states) that each visualize the probability (<=0) of their state being the ancestral state at a given node. In the legend, all states have equal probability to indicate all colours/states.

The likelihood of states of trap characters at nodes throughout the phylogenetic tree is shown in Fig. 18. According to this reconstruction, UVTT derived from UPTT. Species investigated of the UPTT are terrestrial, (facultative) epiphytic, or (facultative) lithophytic and their traps possess – except for the acute-angled trapdoors – most of the structural-functional features as described for the UVTT (short entrances, trapdoors with – yet not distinct – concentric constrictions which perform curvature inversions prior to opening, and cavities). It remains to be investigated how the free door edge is fastened on the threshold in an obtuse angle. Probably, large amounts of mucilage aid in securing the door.

Species of UUTT, UVTT, and UPTT are characterized by traps that perform suction after triggering and spontaneously. Apart from this general similarity, the trapdoor movements after triggering are quite different. In *U*. subg. *Utricularia* (aquatic UVTT species and terrestrial UPTT species), triggering entails curvature inversions of the doors, which open afterwards. It is speculated that trap triggering is based on such a mechanical instability mechanism^13,17^. As the pressure difference between the trap inside and outside rises due to continuous pumping of water out of the trap^26^, the trapdoor becomes more and more sensitive to mechanical perturbations. Trigger hairs protruding from the door are assumed to act as levers, transmitting the slightest deformation onto the trapdoor that then progressively deforms. The door inverts its curvature, channelled by the cellular constrictions, and then opens. Probably, narrow angled doors in tubular trap entrances (UPTT, UUTT) with large amounts of mucilage for sealing and fastening the oblique door are less susceptible to such mechanical disturbances, which might be an advantage when the traps are not surrounded constantly by water. It would be interesting to investigate the mechanical sensitivity among the different trap door movement types in consecutive studies, as differential mechanical sensitivity is associated with variation in evolutionary rate in biomechanical systems^27^.

Non-aquatic bladderworts face irregular (e.g., daily or seasonally changing) water availability as well as physical barriers by substrate. Accordingly, a more protected entrance (i.e. due to appendages and smaller size) and sessile glands instead of protruding trigger hairs are presumably of advantage regarding trapdoor functionality and suspected water retention capability. In species without trigger hairs, it is conceivable that sessile glands act like trigger hairs. Due to the small size, compared to the very long trigger hairs that presumably act as levers, the ‘range for triggering’ of the trapdoors is reduced. This could be an advantage in non-aquatic habitats. The conspicuous pads on the trapdoors of *U. warburgii* and *U. uniflora* (Figs 7B, 8E) and the free door edge with teeth in *U. uniflora* (Fig. 8C,E,F) are of unknown function. We can only hypothesize that they might influence trapdoor fastening on the threshold.

It would be interesting to see if rheophytic *Utricularia* species feature other trapdoor movement types, or if they show similarity with any of the known trapdoor types. Although Lloyd^15,28^ mentioned that the traps of the rheophyte *U. neottioides* of *U*. sect*. Avesicaria* perform suction, they are very rarely developed, and often these plants are entirely devoid of traps^5,29^. However, as the rheophytes of *U*. sect. *Avesicaria* and *Avesicarioides* derived from two lineages of terrestrial members of *U*. subg. *Utricularia* and *U*. subg. *Bivalvaria*, respectively^8,9^, we assume that their rarely formed traps are of the UPTT (*U*. sect. *Avesicaria*) and/or UUTT (*U*. sect. *Avesicarioides*).

Top fluid speeds during suction are similar among the three species investigated, each representing a main trapdoor type with similarly sized traps (*U. gibba* = UVTT, *U. praelonga* = UPTT, *U. prehensilis* = UUTT). *U. gibba* exhibits highest suction duration as well as the largest aspiration zone. *U. prehensilis* and *U. praelonga* are almost identical to each other in these premises. The high fluid velocity generated by the traps most presumably diminishes the full development of a fluid boundary layer. Thus, losses due to friction are reduced because little shearing stresses occur. The remaining losses within the system are due to inertia effects making the suction of *Utricularia* very efficient. It has still to be analysed which features contribute to the observed differences regarding aspirated volume. It can be speculated that it might be caused by differences in trap wall stiffness between the various species (cf. ref.^25^). It has to be noted – based on our experimental experience – that aquatic species are generally much more easy to trigger manually than non-aquatics ones and that the presented results do not necessarily depict suction behaviour at maximum deflation. Comparative prey capture analyses of traps of non-aquatic species could help assessing the effectiveness of such small suction devices (cf. ref.^30^).

The discussed relations between trap functioning, functional trap morphology, and evolutionary lineages within the genus *Utricularia* yield information on how bladderworts could have evolved the greatest species diversity within carnivorous plants. Possible key innovations (e.g., suction) may have resulted in novel phenotypes facilitating the establishment of new habitats and thus amplified the morphological diversity in *Utricularia*. Such saltational evolutionary innovations have been proposed to play a crucial role regarding the vegetative morphology in Lentibulariaceae^31^.

## Materials and Methods

### Source and cultivation of plants for morphological and biomechanical analyses

Most species investigated (Table 1) were purchased from Gartenbau Thomas Carow (Nüdlingen, Germany) and cultivated in the greenhouses of the Botanic Garden Freiburg. *U. multifida* and *U. menziesii* were obtained as seed from commercial source (Allen Lowrie, Perth) and cultivated by A.F.

### Morphological analyses

Preparation for SEM (LEO 435 VP, Leica, Wiesbaden, Germany) involved successive dehydration in methanol (30%, 70%, 99,9%, each step for 2–3 days) or rapid methanol fixation^32^, critical-point drying (LPD 030, Bal-Tec/Leica Mikrosysteme Vertrieb GmbH, Wetzlar, Germany) and gold-sputtering (Sputter Coater 108 auto, Cressington Scientific Instruments Ltd., Watford, England UK).

Preparation for LM involved fixation (90 parts 50% isopropanol and 10 parts 99.5% glycerine) and infiltration with Technovit 7100 (Heraeus Kulzer GmbH, Hanau, Germany). The material was sectioned on a sliding microtome at 10 µm thickness. Sections were stained with 10% aqueous toluidine blue (Chroma-Incorporation, Stuttgart, Germany) and alternatingly rinsed with deionized water and 100% isopropanol. The microscopy slides were sealed with Entellan (Merck, Darmstadt, Germany). A BX61 automated light microscope (Olympus Life Science Corp., Hamburg, Germany) equipped with a DP71 digital camera and the software Cell-P 2.8 (Olympus Soft Imaging Solutions) were used.

### Analyses of door kinematics and suction dynamics

Traps were recorded from lateral positions with a high-speed camera (Motion Scope Y4, Redlake, USA) (recording speed: 10,000 fps) in combination with a stereo microscope (Olympus SZX9 or SZX7, Olympus Corp., Tokyo, Japan) and a Constellation 120 high-performance LED light source (IDT Inc., Tallahassee, Florida, USA). The software Motion Studio 2.08.03 (IDT, Tallahassee, USA) was used for data acquisition.

Subterraneous trap-bearing stolons were carefully dissected from the plants and transferred into petri dishes filled with rainwater. When the bladders were ready to fire (indicated by concave trap walls), they were manually triggered by touching the trigger hairs or the outer trapdoor surface (in species without trigger hairs) by using human hair. A laser sheet technique^17^ could not be applied to visualize the motion of the median trapdoor axis due to the small size of the doors in the species investigated as well as to morphological impediments (appendages at the trap entrances). Therefore, the frames acquired from high-speed cinematography were processed in Fiji/ImageJ^33^, and by adjusting brightness and contrast the kinematics of the trapdoors visualized. Our terminology of trapdoor movement types (see Results) is according to nomenclature used for the species where the respective movement was first observed.

Suction dynamics were analysed in representatives of the three main trapdoor movement types: *U. gibba* (aquatic) (numbers of traps studied n = 9), *U. praelonga* (terrestrial) (n = 6) and *U. prehensilis* (terrestrial) (n = 6), with traps similar in length (ca. 1 mm). The fluid flow could be quantified in Fiji/ImageJ by tracking hollow glass spheres of 2–20 µm in size and of 1.1 g cm^−3^ in density (Polysciences, Inc., Washington, USA) which were carefully added to the water in the vicinity of the trap entrance. Particles could be tracked until they entered the trap entrance where they were obscured by the lateral walls. The particle farthest from the trap entrance being successfully trapped determined the boundary of the aspiration zone. Normal distribution of the values measured for the individual particles (Shapiro-Test) allowed for pooling of particle data for the individual species. Reynold’s numbers were calculated using mean values of particle velocities of individuals and the diameter of the trap entrances, based on the assumption of a water density of 1000 kg m^−^³ and a dynamic viscosity of 1.0020 mPa*s at 20 °C. Statistical analyses were performed using a Kruskal-Wallis-Test. All statistical tests were performed with an alpha value set to 5%.

### Analyses of spontaneous firings

Dissected trap-bearing stolons were submersed in petri dishes with rainwater and filmed for up to 15 h using a SZX9 Olympus stereo microscope with a Colorview Soft Imaging System camera (Olympus Life Science Inc., Hamburg, Germany; recording speed: 1 frame per 5 min) and the software Cell-D 2.6 (Olympus Soft Imaging Solutions). Data processing was performed with Fiji, MS Excel 2010 (Microsoft).

### Phylogenetic reconstruction

Two different molecular datasets were compiled: (i) For a chloroplast *trnK/matK* dataset, we combined sequence data from ref.^9^ with sequences newly generated for this study, representing a total of 105 species of the genus *Utricularia* plus five outgroup taxa (Table S1). This data matrix includes nearly 50% of the species known from this genus, and samples 26 out of 32 sections delimited by refs^5,9^. The dataset also includes the recently described *U*. section *Minutae* ^34^, which has been shown to fall within *U*. sect. *Enskide* of *U*. subg. *Bivalvaria* ^22^.

(ii) Sequences from plastid *trnK*/*matK* and *trnL-F* region were combined for a second dataset comprising 79 taxa from *Utricularia* plus five outgroup taxa from Lamiales. The existing *trnL-F* sequences deposited in GenBank had some issues (e.g. 11 ambiguities in 845 nt in AF482666 [*U. oliveriana*] compared to our new sequence), so we decided to re-sequence this region for all taxa as far as plant material was available. Most plants sequenced in the current study were cultivated in the Botanic Gardens of Bonn University or in the private collection of A.F. Detailed information on plant material, the respective vouchers and GenBank accessions are given in Table S1.

### Amplification, sequencing and phylogenetic alignment

Total genomic DNA was isolated either following the modified CTAB protocol described in ref.^35^, or using the AVE Gene Plant Genomics DNA Mini Kit (AVE Gene, Korea). As phylogenetic markers, the *trnK* intron including the *matK*-gene, and *trnL-F* region were amplified using standard PCR. Primers used for amplification and sequencing are given in ref.^36^. Reactions were performed in 50 µl volumes containing 2 µl template DNA (10 ng/µl), 10 µl dNTP mix (1.25 mM each), 2 µl of each forward and reverse primer (20 pm/µl) and 0.3 µl Taq polymerase (5 U/µl, Peqlab). Thermal cycling was performed on a Biometra T3 thermocycler (Biometra Ltd., Germany) using the following PCR profiles: 1:30 min at 96 °C, 1 min at 50 °C, 1:30 min at 72 °C, 35 cycles of 30 secs at 96 °C, 1 min at 50 °C, 1:30 min at 72 °C, and a final extension time of 10 min at 72 °C for *trnK* intron; 35 cycles of 1 min at 94 °C, 1 min at 52 °C and 2 min at 72 °C, followed by a final extension time of 15 min at 72 °C for *trnL-F* region. Fragments were gel-purified on a 1.2% agarose gel (Neeo-agarose, Roth, Germany), extracted with the Gel/PCR DNA Fragments Extraction Kit (AVE Gene, Korea) and directly sequenced on an ABI3730XL automated sequencer at Macrogen sequencing service (Macrogen Inc., Seoul, Korea).

Both *trnK*/*matK* and *trnL-F* sequences showed frequent length mutations, even the *matK* coding region included numerous insertions and deletions. Since all current alignment software was found to not accurately account for the nature and frequency of microstructural changes in these non-coding plastome regions, the nucleotide sequences were aligned manually with the alignment editor PhyDE (http://www.phyde.de). Indels were coded according to the simple indel coding method (SIC)^37,38^.

### Tree inference and evaluation

For maximum parsimony (MP) reconstruction, the search for shortest trees was performed using the parsimony ratchet analysis as implemented in PRAP2^39^ with the following settings: 200 ratchet replicates with 25% of characters reweighted by 2; 10 random addition cycles. A strict consensus of the shortest trees was constructed. For tree evaluation 10,000 bootstrap replicates were calculated, holding only one tree in memory. For maximum likelihood (ML) analyses, RAxML v7^40^ was used. During the search for the best tree, the GTRGAMMA model was used, while GTRCAT was employed during the 500 bootstrap replicates. Bayesian inference of phylogeny (BI) was done in MrBayes V3^41^ with the general time reversible model of nucleotide substitution, assuming different stationary nucleotide frequencies and site specific rate categories for each codon position. Posterior probabilities of parameters were estimated by sampling from the posterior probability distribution approximated via Metropolis-coupled Markov chain Monte Carlo. The temperature of the heated chain was set to 0.2. Chains were sampled every 100 generations. Two runs, each with four chains were run for 2,000,000 generations, starting with random trees. A strict consensus tree was calculated from trees sampled after a burn-in of 10% of the MCMC generations. Support values from all types of analysis were mapped on the tree topology from the ML analysis and conflicting nodes were identified with help of TreeGraph2^42^.

### Ancestral state reconstruction

BayesTraits V2^43^ was used to infer ancestral states of trap morphological characters. A reduced tree was used that incorporates only those taxa for which trap morphological characters were assembled. In this tree, sections were represented by the same species for which trap data were compiled, except in the case of *U*. sect. *Aranella* (*U. parthenopipes*) and *U*. sect. *Utricularia* (*U. macrorhiza*), for which the closest relative included in the molecular phylogenetic dataset was used. Branch lengths in the reduced tree were optimized via ML using the same model as described above, and this tree was subjected to BayesTrait’s multistate program, along with characters encoded according to Table S1 and a BayesTraits command file generated with help of TreeGraph2. BayesTrait’s generated log files were parsed by TreeGraph2 to create pie charts that show the relative probabilities of states at all tree nodes. Prior to encoding, the character ‘trapdoor angle’ was categorized into four classes.

### Data availability statement

The datasets generated during and analyzed during the current study are available from the corresponding author on reasonable request.

## Electronic supplementary material

Movie S1

Movie S2

Movie S3

Movie S4

Movie S5

Movie S6

Movie S7

Movie S8

Movie S9

Legends for Movies S1-S9

Table S1
