# Supplementary material for: Trap diversity and character evolution in carnivorous bladderworts (Utricularia, Lentibulariaceae)
Source: Sci Rep. 2017 Sep 21;7:12052. doi: 10.1038/s41598-017-12324-4 (PMC5608911; doi:10.1038/s41598-017-12324-4)
Supplement: Supplementary file 10 — Legends for Movies S1-S9 [file 41598_2017_12324_MOESM10_ESM.pdf]

SUPPLEMENTARY INFORMATION FOR

Trap diversity and character evolution in carnivorous bladderworts (*Utricularia*, Lentibulariaceae)

Anna Sofia Westermeier[1,2], Andreas Fleischmann[3,4], Kai Müller[5], Bastian Schäferhoff[5,6], Carmen Rubach[1], Thomas Speck[1,2], Simon Poppinga[1*]

[1]*Plant Biomechanics Group, Botanic Garden, University of Freiburg, Schänzlestraße 1, D-79104 Freiburg im Breisgau, Germany.*

[2]*Freiburg Center for Interactive Materials and Bioinspired Technologies (FIT), University of Freiburg, Georges-Köhler-Allee 105, D-79110 Freiburg im Breisgau, Germany.*

[3]*Botanische Staatssammlung München, Menzingerstraße 67, D-80638 München, Germany.*

[4]*GeoBio-Center LMU, Center of Geobiology and Biodiversity Research, Ludwig-Maximilians-University, München, Germany.*

[5]*Westfälische Wilhelms-Universität Münster, Institut für Evolution und Biodiversität, AG Evolution und Biodiversität der Pflanzen, Hüfferstraße 1, D-48149 Münster, Germany.*

[6]*present address: PAN Institut für Endokrinologie und Reproduktionsmedizin, Zeppelinstraße 1, D-50667 Köln, Germany*

[*]*Corresponding author. Email: simon.poppinga@biologie.uni-freiburg.de, telephone: 0049-(0)761-203-2999*

Legends for Supplementary Movies

Trapdoor movement

Movie S1: Trapdoor movement and suction of tracer particles in *U. gibba* (UVTT 1), recording speed: 10,000 fps, playback rate: 20 fps.

Movie S2: Trapdoor movement of *U. resupinata* (UVTT 2), recording speed: 10,000 fps, playback rate: 20 fps.

Movie S3: Trapdoor movement of *U. praelonga* (UPTT), recording speed: 10,000 fps, playback rate: 20 fps.

Movie S4: Trapdoor movement of *U. uniflora* (UUTT 1), recording speed: 10,000 fps, playback rate: 20 fps.

Movie S5: Trapdoor movement of *U. menziesii* (UUTT 2), recording speed: 10,000 fps, playback rate: 20 fps.

Movie S6: Trapdoor movement of *U. warburgii* (UUTT 3), recording speed: 10,000 fps, playback rate: 20 fps.

Movie S7: Trapdoor movement of *U. welwitschii* (UUTT 4), recording speed: 10,000 fps, playback rate: 20 fps.

Suction dynamics

For *U. gibba*, please see Video S1.

Movie S8: Suction of tracer particles in *U. prehensilis*, recording speed: 10,000 fps, playback rate: 20 fps.

Movie S9: Suction of tracer particles in *U. praelonga*, recording speed: 10,000 fps, playback rate: 20 fps.
