# Supplementary material for: Trap diversity and character evolution in carnivorous bladderworts (Utricularia, Lentibulariaceae)
Source: Sci Rep. 2017 Sep 21;7:12052. doi: 10.1038/s41598-017-12324-4 (PMC5608911; doi:10.1038/s41598-017-12324-4)
Supplement: Supplementary file 11 — Table S1 [file 41598_2017_12324_MOESM11_ESM.pdf]

SUPPLEMENTARY INFORMATION FOR

Trap diversity and character evolution in carnivorous bladderworts (*Utricularia*, Lentibulariaceae)

Anna Sofia Westermeier[1,2], Andreas Fleischmann[3,4], Kai Müller[5], Bastian Schäferhoff[5,6], Carmen Rubach[1], Thomas Speck[1,2], Simon Poppinga[1*]

[1]Plant Biomechanics Group, Botanic Garden, University of Freiburg, Schänzlestraße 1, D-79104 Freiburg im Breisgau, Germany.

[2]Freiburg Center for Interactive Materials and Bioinspired Technologies (FIT), University of Freiburg, Georges-Köhler-Allee 105, D-79110 Freiburg im Breisgau, Germany.

[3]Botanische Staatssammlung München, Menzingerstraße 67, D-80638 München, Germany.

[4]GeoBio-Center LMU, Center of Geobiology and Biodiversity Research, Ludwig-Maximilians-University, München, Germany.

[5]Westfälische Wilhelms-Universität Münster, Institut für Evolution und Biodiversität, AG Evolution und Biodiversität der Pflanzen, Hüfferstraße 1, D-48149 Münster, Germany.

[6]present address: PAN Institut für Endokrinologie und Reproduktionsmedizin, Zeppelinstraße 1, D-50667 Köln, Germany

[*]Corresponding author. Email: simon.poppinga@biologie.uni-freiburg.de, telephone: 0049-(0)761-203-2999

Table S1: Plant material used for the molecular phylogenetic study. Given are species names and taxonomic classification according to ref. 5 (see main article). If previously published sequence data was used, only the GenBank accession number is given, otherwise voucher information is additionally shown.

Species	**Section**	***trnK/matK***	***trnLF***
***Utricularia adpressa* Salzm. Ex A.St.Hil. et Girard**	*Oligocista*	MF765548; A. Fleischmann s.n., Venezuela, Bolivar, Gran Sabana, cult. Fleischmann	AF482628
***Utricularia alpina* Jacq.**	*Orchidioides*	AF531822	MF765573; Kai Müller KM 712 (BONN), BG Bonn 15081
***Utricularia amethystina* Salzm. ex A.St.-Hil et Girard**	*Foliosa*	MF765546; A.Fleischmann s.n. (M), Venezuela, Estado Bolívar, Mt. Kukenán-tepui	MF765576; A.Fleischmann s.n. (M), Venezuela, Estado Bolívar, Mt. Kukenán-tepui
***Utricularia arcuata* Wight**	*Oligocista*	MF765518; N. Hobbhahn s.n. (as "*U. purpurascens*"), cult. Fleischmann	-
***Utricularia arenaria* A.DC.**	*Calpidisca*	MF765500; F. Rivadavia s.n. (SPF), South Africa, cult. Fleischmann	MF765592; F. Rivadavia s.n. (SPF), South Africa, cult. Fleischmann
***Utricularia arnhemica* P.Taylor**	*Pleiochasia*	MF765494; A. Lowrie s.n. (Herb Low Perth), Australia, Arnhem Land,Queensland, cult Fleischmann	MF765600; A. Lowrie s.n. (Herb Low Perth), Australia, Arnhem Land,Queensland, cult Fleischmann
***Utricularia asplundii* P.Taylor**	*Orchidioides*	MF765551; S.Vieira s.n., cult. Fleischmann, Colombia, Satander	AF482631
***Utricularia aureomaculata* Steyerm.**	*Steyermarkia*	MF765532; cult. Fleischmann, Venezuela, Murosipan-tepui	MF765558; cult. Fleischmann, Venezuela, Murosipan-tepui
***Utricularia australis* R.Br.**	*Utricularia*	AF531823	AF482633
***Utricularia babui* Yadav, Sardesai & Gaikwad**	*Oligocista*	MF765516; A. Fleischmann & P. Suksathan s.n. (M, QBC), Thailand, Chiang Mai Province, Doi Inthanon, cult. Fleischmann	-
***Utricularia benjaminiana* Oliv.**	*Utricularia*	MF765539; F. Rivadavia & A. Fleischmann Z46 (M), Zambia, Lake Chila	MF765568; F. Rivadavia & A. Fleischmann Z46 (M), Zambia, Lake Chila
***Utricularia bifida* L.**	*Oligocista*	MF765522; H. Schäfer s.n. (M), Australia, N-Queensland, Fruit Bat Falls, cult. Fleischmann	-
***Utricularia biloba* R.Br.**	*Nelipus*	MF765534; B. Schäferhoff s.n. (BONN), BG Bonn 19853	MF765560; B. Schäferhoff s.n. (BONN), BG Bonn 19853
***Utricularia bisquamata* Schrank**	*Calpidisca*	MF765503; B. Schäferhoff s.n., (BONN), BG Bonn s.n.	MF765591; B. Schäferhoff s.n., (BONN), BG Bonn s.n.
***Utricularia blanchetii* A.DC.**	*Aranella*	AF531841	MF765597; Kai Müller KM 704 (BONN), BG Bonn 17170
***Utricularia bremii* Heer ex Kölliker**	*Utricularia*	MF765536; B. Schäferhoff s.n. (BONN), BG Bonn 2241	MF765563; B. Schäferhoff s.n. (BONN), BG Bonn 2241
***Utricularia breviscapa* Wright ex Grieseb.**	*Utricularia*	MF765537; B. Schäferhoff s.n. (BONN), BG Bonn s.n.	MF765565; B. Schäferhoff s.n. (BONN), BG Bonn s.n.
***Utricularia caerulea* L.**	*Nigrescentes*	MF765507; A. Fleischmann et al. s.n. (M), Thailand, Trang Peninsular Botanical Garden, cult. Fleischmann	AF482636
***Utricularia calycifida* Benj.**	*Psyllosperma*	AF531824	MF765578; Kai Müller KM 705 (BONN), BG Bonn 14514
***Utricularia capilliflora* F.Muell.**	*Pleiochasia*	MF765495; A. Lowrie s.n. (Herb Low Perth), Australia, Wickham Point, cult Fleischmann	MF765603; A. Lowrie s.n. (Herb Low Perth), Australia, Wickham Point, cult Fleischmann
***Utricularia chrysantha* R.Br.**	*Enskide*	MF765527; A. Fleischmann s.n.	MF765589; A. Lowrie s.n., Australia, Northern Territory, Noonamah, cult. Fleischmann
***Utricularia cornuta* Michx.**	*Stomoisia*	MF765524; A. Darr, B. Pittman & W. Pittman 2465 (M), USA, SC, Pickens County, Cedar Rock Mountain in Nine Times area	AF482638
***Utricularia dichotoma* Labill.**	*Pleiochasia*	AF531826	MF765601; Kai Müller KM 714 (BONN), BG Bonn 16419
Utricularia dunlopii	*Pleiochasia*	MF765493; A. Lowrie s.n. (Herb Low Perth),	-

**P.Taylor**		Australia, N.T., cult Fleischmann	
***Utricularia endressii* Rchb.f.**	*Orchidioides*	MF765543; B. Schäferhoff 30 (BONN), BG Bonn s.n.	AF482642
***Utricularia erectiflora* A.St.Hil. et Girard**	*Oligocista*	MF765514; A. Fleischmann s.n., Brazil, Amazonas State, Dept. Roraima Dist. Pakaraima, cult. Fleischmann	AF482643
***Utricularia firmula* Welw. Ex Oliv.**	*Calpidisca*	MF765501; F. Rivadavia & A. Fleischmann Z81 (M), Zambia, Central Province, 16km S of Kapiri Mposhi	-
***Utricularia flaccida* A.DC.**	*Setiscapella*	AF531830	AF482644
***Utricularia floridana* Nash**	*Utricularia*	MF765541; B. Schäferhoff s.n. (BONN), BG Bonn s.n.	MF765566; B. Schäferhoff s.n. (BONN), BG Bonn s.n.
***Utricularia foliosa* L.**	*Utricularia*	MF765538; F. Rivadavia & A. Fleischmann Z68 (M), Zambia, Lake Lusiwasi	MF765564; F. Rivadavia & A. Fleischmann Z68 (M), Zambia, Lake Lusiwasi
***Utricularia foveolata* Edgew.**	*Oligocista*	AF531850	AF482645
***Utricularia fulva* F.Muell.**	*Enskide*	MF765526; B. Schäferhoff s.n. (BONN), BG Bonn 2654	MF765590; B. Schäferhoff s.n. (BONN), BG Bonn 2654
***Utricularia geminiloba* Benj.**	*Iperua*	MF765545; B. Schäferhoff s.n. (BONN), BG Bonn 2251	MF765569; B. Schäferhoff s.n. (BONN), BG Bonn 2251
***Utricularia graminifolia* Vahl**	*Oligocista*	MF765517; A. Fleischmann s.n., coll. P. Debbert	AF482649
***Utricularia heterochroma* Steyerm.**	*Mirabiles*	MF765533; A. Fleischmann s.n., Venezuela, Etdo. Bolívar, Disto. Piar, Macizo del Chimantá, Amurí-tepui	-
***Utricularia hirta Klein ex* Link**	*Meionula*	MF765509; C. Klein s.n., Thailand, cult. Fleischmann	-
***Utricularia hispida* Lam.**	*Psyllosperma*	AF531829	MF765579; Kai Müller KM 716 (BONN), BG Bonn 17176
***Utricularia humboldtii* Schomb.**	*Iperua*	AF531836	MF765571; Kai Müller KM 717 (BONN), BG Bonn 4839
***Utricularia intermedia* Hayne**	*Utricularia*	AF531839	AF482651
***Utricularia involvens* Ridl.**	*Oligocista*	MF765550; B. Schäferhoff s.n. (BONN), BG Bonn 25234	-
***Utricularia jamesoniana* Oliv.**	*Orchidioides*	MF765547; S.Vieira s.n., cult. Fleischmann, Colombia	MF765574; S.Vieira s.n., cult. Fleischmann, Colombia
***Utricularia juncea* Vahl**	*Stomoisia*	AF531832	MF765587; Kai Müller KM 746 (BONN), USA
***Utricularia kamienskii* F-Muell.**	*Pleiochasia*	MF765499; A. Lowrie s.n., Australia, Girraween, N.T., cult Fleischmann	MF765602; A. Lowrie s.n., Australia, Girraween, N.T., cult Fleischmann
***Utricularia kimberleyensis* C.A.Gardn**	*Pleiochasia*	MF765492; A. & H. Hennern 002 (M), Australia, Western Australia, Kimberleys, Mornington Station, cult. Fleischmann	-
***Utricularia lateriflora* R.Br.**	*Australes*	MF765504; A. Lowrie s.n., Australia, Tasmania, Blackman´s Bay, cult. Fleischmann	AF482654
***Utricularia laxa* A.St.Hil.**	*Oligocista*	MF765521; B. Schäferhoff s.n. (BONN), BG Bonn s.n.	MF765585; B. Schäferhoff s.n. (BONN), BG Bonn s.n.
***Utricularia leptorhyncha* O.Schwarz**	*Pleiochasia*	MF765498; A. Lowrie 3198 (Herb Low Perth), Australia, Berry Springs, N.T.	-
***Utricularia livida* E.Meyer**	*Calpidisca*	AF531833	AF482655
***Utricularia lloydii* Merl ex F.E.Lloyd**	*Oligocista*	MF765549; A. Fleischmann s.n., Venezuela, Bolivar, Gran Sabana, cult. Fleischmann	MF765586; A. Fleischmann s.n., Vebezuela, Bolivar, Gran Sabana, cult. Fleischmann
***Utricularia longifolia* Gardner**	*Psyllosperma*	AF531834	MF765509; Kai Müller KM 718 (BONN), BG Bonn 15120
Utricularia macrorhiza	*Utricularia*	AF531835	AF482657

**Leconte**			
***Utricularia mannii* Oliv.**	*Chelidon*	MF765520; Th. Franke & L. Beenken s.n. (M), Mt. Cameroon, Cameroon, cult. Fleischmann	MF765526; Th. Franke & L. Beenken s.n. (M), Mt. Cameroon, Cameroon, cult. Fleischmann
***Utricularia menziesii* R.Br.**	*Pleiochasia*	MF765558; A. Lowrie & A. Fleischmann s.n., (Herb Low Perth), Australia, Brookton, Western Australia	-
***Utricularia minor* L.**	*Utricularia*	MF765554; B. Schäferhoff s.n. (BONN), BG Bonn 2243	MF765562; B. Schäferhoff s.n. (BONN), BG Bonn 2243
***Utricularia minutissima* Vahl**	*Meionula*	MF765510; A. Fleischmann et al. s.n. (M), Thailand, Trang Peninsular Botanical Garden, cult. Fleischmann	-
***Utricularia moniliformis* P.Taylor**	*Phyllaria*	MF765508; C. Klein s.n., Sumatra, cult Fleischmann	MF765594; C. Klein s.n., Sumatra, cult Fleischmann
***Utricularia multifida* R.Br.**	*Polypompholyx*	AF531848	AF482659
***Utricularia nana* A.St.Hil. & Girard**	*Benjaminia*	AF531837	AF482661
***Utricularia nelumbifolia* Gardn.**	*Iperua*	MF765544; B. Schäferhoff s.n. (BONN), BG Bonn 20395	AF482662
***Utricularia nephrophylla* Benj.**	*Iperua*	AF521827	MF765570; Kai Müller KM 720 (BONN), BG Bonn 17180
***Utricularia nervosa* G.Weber ex Benj.**	*Setiscapella*	MF765552; A. Fleischmann s.n., Venezuela, Gran Sabana	MF765557; A. Fleischmann s.n., Venezuela, Gran Sabana
***Utricularia nigrescens* Sylvén**	*Setiscapella*	MF765529; F. Rivadavia s.n., Brazil, Jalapao, cult. Fleischmann	-
***Utricularia novae-zelandiae* Hook**	*Pleiochasia*	MF765490; B. Schäferhoff 48 (BONN), BG Bonn 22497	-
***Utricularia olivacea* Wright ex Grieseb.**	*Utricularia*	AF531840	MF765567; Kai Müller KM 620 (BONN), USA
***Utricularia oliveriana* Steyerm.**	*Avesicaria*	MF765530; A. Fleischmann s.n., Venezuela, Etdo. Bolívar, Municipio Gran Sabana, cult. Fleischmann	MF765556; A. Fleischmann s.n., Venezuela, Etdo. Bolívar, Municipio Gran Sabana, cult. Fleischmann
***Utricularia parthenopipes* P.Taylor**	*Aranella*	AF531842	MF765596; Kai Müller KM 749 (BONN), BG Bonn 17181
***Utricularia paulineae* Lowrie**	*Pleiochasia*	MF765488; S. Spence s.n., Australia, Western Australia, near Walepole, cult. Fleischmann	MF765599; S. Spence s.n., Australia, Western Australia, near Walepole, cult. Fleischmann
***Utricularia petertaylorii* Lowrie**	*Pleiochasia*	MF765485; A. Lowrie s.n. (Herb Low Perth), Australia, Western Australia, cult. Fleischmann	-
***Utricularia praelonga* A.St.Hil. & Girard**	*Psyllosperma*	AF531843	MF765509; Kai Müller KM 720 (BONN), BG Bonn 17183
***Utricularia praetermissa* P.Taylor**	*Orchidioides*	MF765553; B. Schäferhoff s.n. (BONN), BG Bonn s.n.	MF765572; B. Schäferhoff s.n. (BONN), BG Bonn s.n.
***Utricularia prehensilis* E.Mey.**	*Oligocista*	MF765519; B. Schäferhoff 51 (BONN), BG Bonn 17184	MF765582; B. Schäferhoff 51 (BONN), BG Bonn 17184
***Utricularia pubescens* Sm.**	*Lloydia*	AF531844	MF765593; S. Porembski 3852 (ROST), Ivory Coast
***Utricularia purpurea* Walter**	*Vesiculina*	AF531845	MF765559; Kai Müller KM 647 (BONN), USA
***Utricularia purpureo-caerulea* A.St.Hil. ar Girard**	*Aranella*	MF765511; B. Schäferhoff 50 (BONN), BG Bonn s.n.	AF482670
***Utricularia pusilla* Vahl**	*Setiscapella*	MF765531; P. Debbert s.n., Venezuela, base of Ilu-tepui, cult. Fleischmann	-
***Utricularia quelchii* N.E.Br.**	*Orchidioides*	AF531846	MF765575; Kai Müller KM 722 (BONN), Venezuela
***Utricularia quinquedentata* F.Muell ex P.Taylor**	*Pleiochasia*	MF765496; A. Lowrie s.n. (Herb Low Perth), Australia, Tozers Gap, Queensland, cult Fleischmann	-

***Utricularia reflexa* Oliv.**	*Utricularia*	MF765540; F. Rivadavia & A. Fleischmann Z23 (M), Zambia, 30 km N of Mansa	-
***Utricularia reniformis* A.St.Hil.**	*Iperua*	AF531828	AF482671
***Utricularia resupinata* Greene**	*Lecticula*	MF765542; A.Fleischmann s.n., cult. L. Adamec, cult. Fleischmann, Nicaragua	AF488533
***Utricularia rigida* Benj.**	*Avesicarioides*	AF531838	MF765583; S. Porembski 3860 (ROST), Ivory Coast
***Utricularia sandersonii* Oliv.**	*Calpidisca*	AF531847	AF482672
***Utricularia scandens* Benj.**	*Oligocista*	MF765523; F. Rivadavia & A. Fleischmann Z65 (M), Zambia, Northern Province, 102 km S Mpika, cult. Fleischmann	MF765584; F. Rivadavia & A.Fleischmann Z65 (M), Zambia, Northern Province, 102 km S Mpika, cult. Fleischmann
***Utricularia simmonsii* Conran & Lowrie**	*Minutae*	MF765525; A. Lowrie cult., A. Fleischmann cult., Australia, northern Queensland, Lockhart River	MF765588; A. Lowrie cult., A. Fleischmann cult., Australia, northern Queensland, Lockhart River
***Utricularia simplex* R.Br.**	*Australes*	MF765505; S. Spence s.n., Australia, Victoria, Cheeshunt, cult. Fleischmann	AF482673
***Utricularia simulans* Pilger**	*Aranella*	MF765513; A. Fleischmann s.n., Venezuela, Bolivar, Gran Sabana, cult. Fleischmann	AF482674
***Utricularia singeriana* F.Muell.**	*Pleiochasia*	MF765489; A. Lowrie 3979 (Herb Low Perth), Australia, Kakadu N.P., N.T.	-
***Utricularia spiralis* Sm.**	*Oligocista*	AF531851	MF765581; S. Porembski 3853 (ROST), Ivory Coast
***Utricularia stygia* G.Thor**	*Utricularia*	MF765535; B. Schäferhoff s.n. (BONN), BG Bonn 2242	MF765561; B. Schäferhoff s.n. (BONN), BG Bonn 2242
***Utricularia subulata* L.**	*Setiscapella*	AF531821	AF482676
***Utricularia tenuissima* Tutin**	*Martinia*	MF765512; H. Hertel 36741 (M), Venezuela, Estado Bilovar, Kweikin-ima tepui	MF765595; H. Hertel 36741 (M), Venezuela, Estado Bilovar, Kweikin-ima tepui
***Utricularia terrae-reginae* P.Taylor**	*Pleiochasia*	MF765497; A. Lowrie 3857 (Herb Low Perth), Australia, Biffin Swamp, Queensland	-
***Utricularia tortilis* Welw. ex Oliv.**	*Oligocista*	MF765515; F. Rivadavia & A. Fleischmann Z82 (M), Zambia, 16km S of Kapiri Mposhi, cult. Fleischmann	MF765580; F. Rivadavia & A. Fleischmann Z82 (M, SPF), Zambia, 16km S of Kapiri Mposhi, cult. Fleischmann
***Utricularia tridentata* Sylvén**	*Foliosa*	AF531825	MF765577; Kai Müller KM 725 (BONN), BG Bonn 16930
***Utricularia triloba* Benj.**	*Setiscapella*	MF765528; A. Fleischmann s.n., Venezuela, Estado Bolivar, WP-9-12, cult. Fleischmann	MF765555; A. Fleischmann s.n., Venezuela, Estado Bolivar, WP-9-12, cult. Fleischmann
***Utricularia uliginosa* Vahl**	*Oligocista*	AF531849	AF482679
***Utricularia uniflora* R.Br.**	*Pleiochasia*	MF765491; A. Lowrie s.n. (PERTH), Australia, New South Wales, Waterfall, cult. Fleischmann	-
***Utricularia violacea* R.Br.**	*Pleiochasia*	MF765486; A. Lowrie s.n., Australia, Western Australia, Cape Le Grand, cult. Fleischmann	AF482680
***Utricularia volubilis* R.Br.**	*Pleiochasia*	MF765487; B. Schäferhoff s.n. (BONN), BG Bonn 2240	MF765598; B. Schäferhoff s.n. (BONN), BG Bonn 2240
***Utricularia vulgaris* L.**	*Utricularia*	AF531831	AF482681
***Utricularia warburgii* Goebel**	*Nigrescentes*	MF765506; B. Schäferhoff s.n. (BONN), BG Bonn s.n.	-
***Utricularia welwitschii* Oliv.**	*Calpidisca*	MF765502; F. Rivadavia & A. Fleischmann s.n. (M), Zambia, cult. Fleischmann	-
